# Stereoselective Preparation of (*4S*)-1-Methyl-4-Propyl-L-Proline Commencing from (*cis*)-4-Hydroxy-L-Proline

**DOI:** 10.3390/M2003

**Published:** 2025-05-05

**Authors:** Gour Hari Mandal, Shifali Choudhary, Steven P. Kelley, Shyam Sathyamoorthi

**Affiliations:** 1 Department of Medicinal Chemistry, University of Kansas, Lawrence, KS 66047, USA;; 2 Department of Chemistry, University of Missouri—Columbia, Columbia, MO 65211, USA;

**Keywords:** amino acids, antibiotic analogs, stereoselective synthesis

## Abstract

We present a recipe for the stereoselective conversion of commercial (*cis*)-4-hydroxy-L-proline into (*4S*)-1-methyl-4-propyl-L-proline, an analog of the amino acid fragment found in the clinically used antibacterial antibiotic lincomycin. The single-crystal X-ray diffraction analysis of the final target’s hydrochloride salt confirms its identity and absolute stereochemistry.

## 1. Introduction

Due to the rapid rise in bacterial resistance to clinically used natural product antibiotics [1,2], the preparation of non-natural analogs has become a vital pursuit [3–7]. Our laboratory has a programmatic focus on the development of new organic reactions to simplify the syntheses of anti-infective natural and non-natural compounds [8–11]. In line with this agenda, we were attracted to the lincosamide family of antibiotics, whose members include the clinically used lincomycin and clindamycin [12]. Because of their useful activity against Gram-positive bacterial pathogens [13], naturally occurring lincosamides continue to inspire the development of non-natural analogs for future clinical and agricultural use [6,14].

**Figure 1.**
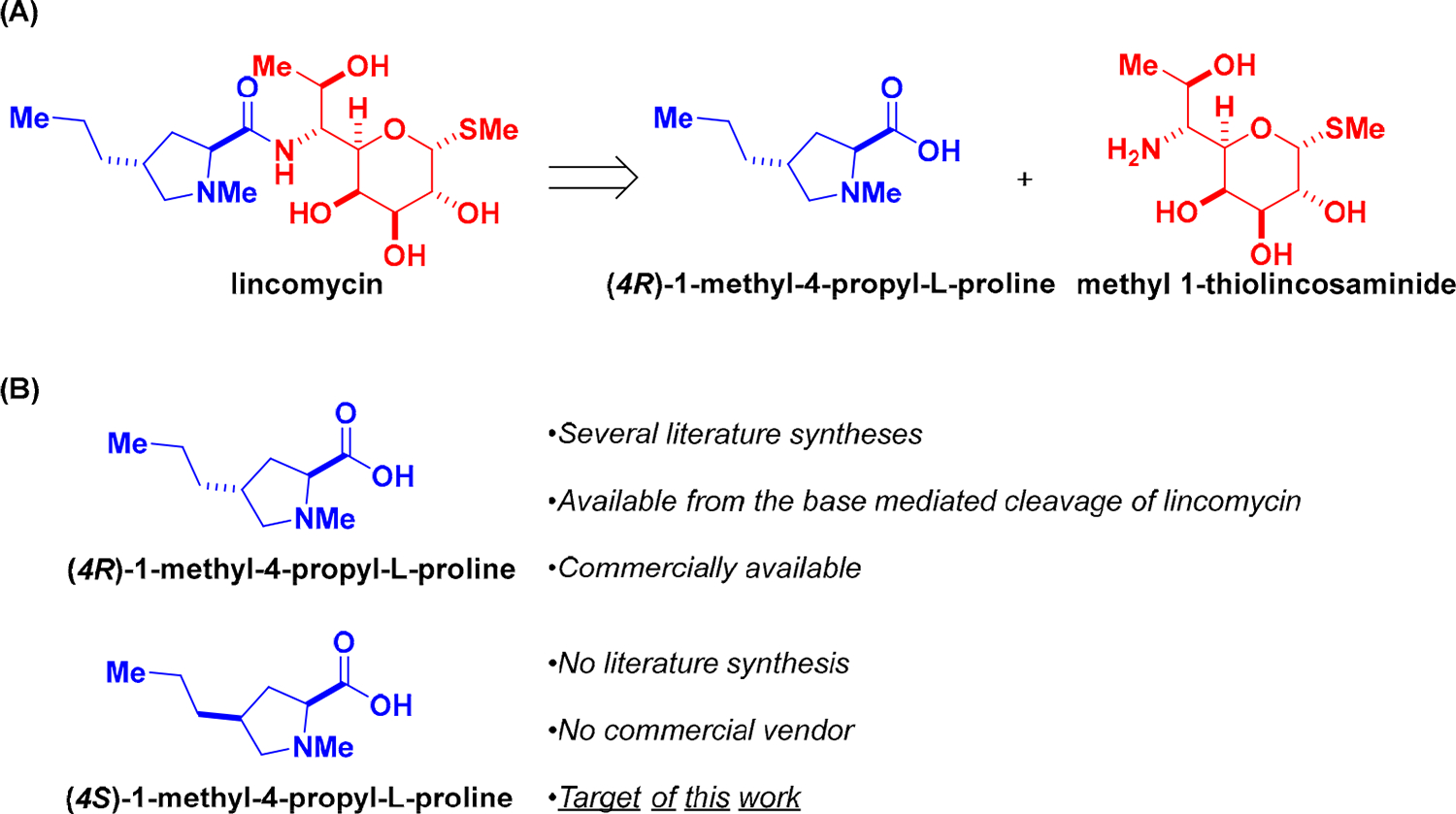
(**A**) Lincomycin is a clinically used natural product antibacterial antibiotic and comprises two smaller natural products. (**B**) The focus of this work is the stereoselective preparation of (4*S*)-1-methy1-4-propyl-L-proline, an analog of the amino acid fragment of lincomycin.

Lincomycin, a natural product and the flagship member of the lincosamides, is a fusion of two smaller compounds: (*4R*)-1-methyl-4-propyl-L-proline, an amino acid, and methyl 1-thiolincosaminide, an *aza*-monosaccharide (Figure 1A). (*4R*)-1-methyl-4-propyl-L-proline is commercially sold and can be obtained from the base-mediated hydrolysis of lincomycin [15–18]. It has served as the target of an elegant synthesis [19]. In sharp contrast, its epimer, (*4S*)-1-methyl-4-propyl-L-proline is not commercially available, and no literature syntheses exist. In this communication, we present a stereoselective preparation of (*4S*)-1-methyl-4-propyl-L-proline from the commercial and widely available (*cis*)-4-hydroxy-L-proline (Figure 1B).

## 2. Results and Discussion

**Figure 2.**
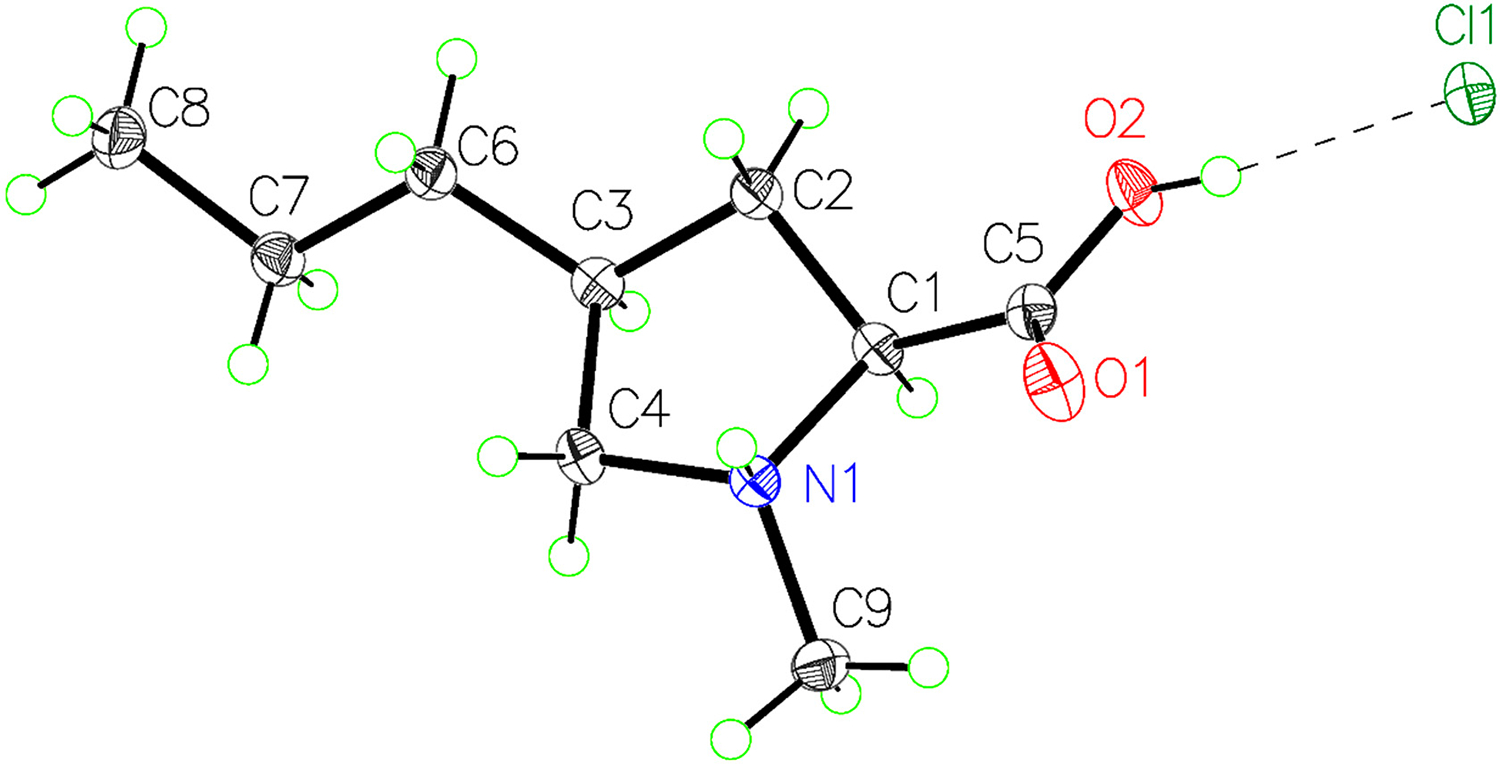
Labeled, 50%-probability, ellipsoid plot of the formula unit of (*4S*)-1-methyl-4-propyl-L-proline hydrochloride (CCDC 2440605).

**Scheme 1.**
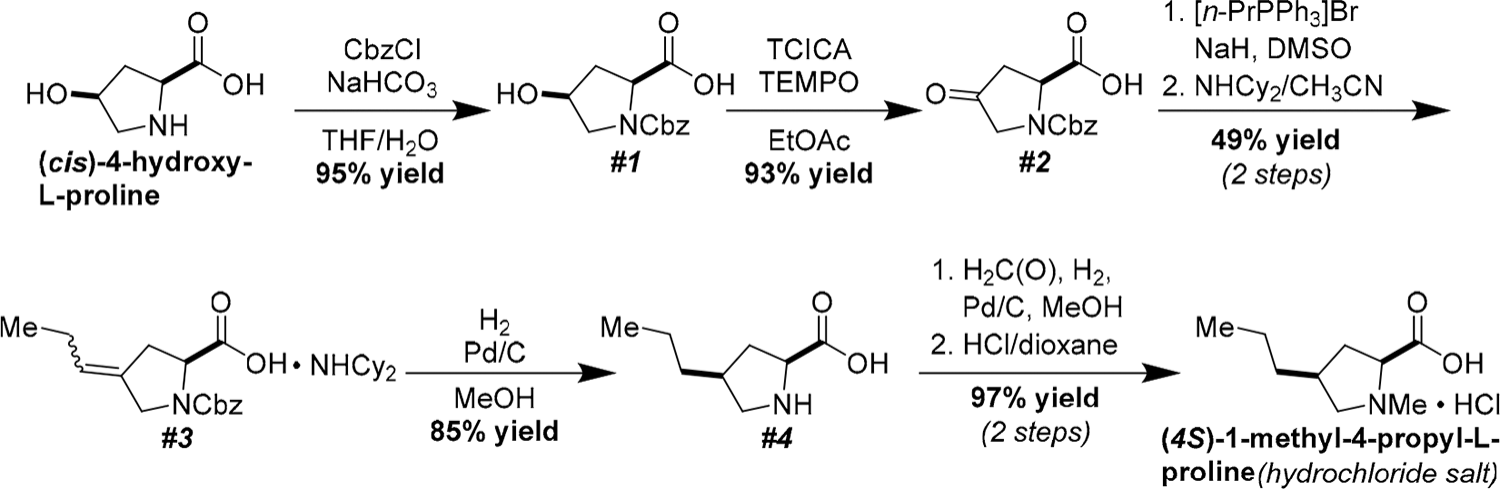
Conversion of commercial (*cis*)-4-hydroxy-L-proline into the hydrochloride salt of (4*S*)-1-methy1-4-propyl-L-proline.

Our synthesis of (*4S*)-1-methyl-4-propyl-L-proline hydrochloride started with attachment of a carboxybenzyl group to the nitrogen atom of commercial (*cis*)-4-hydroxy-L-proline (Scheme 1) [20]. The alcohol in **1** was oxidized to the corresponding ketone using a combination of trichloroisocyanuric acid (TCICA) and TEMPO in ethyl acetate [20]. Wittig olefination proceeded by heating **2** with a combination of propyltriphenylphosphonium bromide and sodium hydride in dimethyl sulfoxide (DMSO) [18]. Other common Wittig conditions, such as using sodium hydride in DMF, were much less successful. While the carboxylic acid was unstable during purification by silica gel chromatography or reversed-phase high-performance liquid chromatography, the dicyclohexylamine salt could be purified by repeated washes with diethyl ether [18]. The hydrogenation of the olefin using palladium on carbon (Pd/C) and 1 atmosphere of hydrogen gas yielded **4** with excellent diastereoselectivity. The high selectivity of this transformation can be explained using a simple steric argument; the palladium hydride species prefers to attack the less hindered face of the olefin, placing the propyl and carboxylic acid groups *syn* to each other. Finally, *N*-methylation proceeded smoothly using formalin, Pd/C, and 1 atmosphere of hydrogen gas [19]. Treatment of the product with a solution of hydrochloric acid in dioxane and washing the resulting salt with diethyl ether yielded (*4S*)-1-methyl-4-propyl-L-proline as its hydrochloride salt. Crystallization of this compound from an ethanolic solution gave single crystals suitable for X-ray diffraction analysis, confirming its identity and absolute stereochemistry (Figure 2).

**Figure 3.**
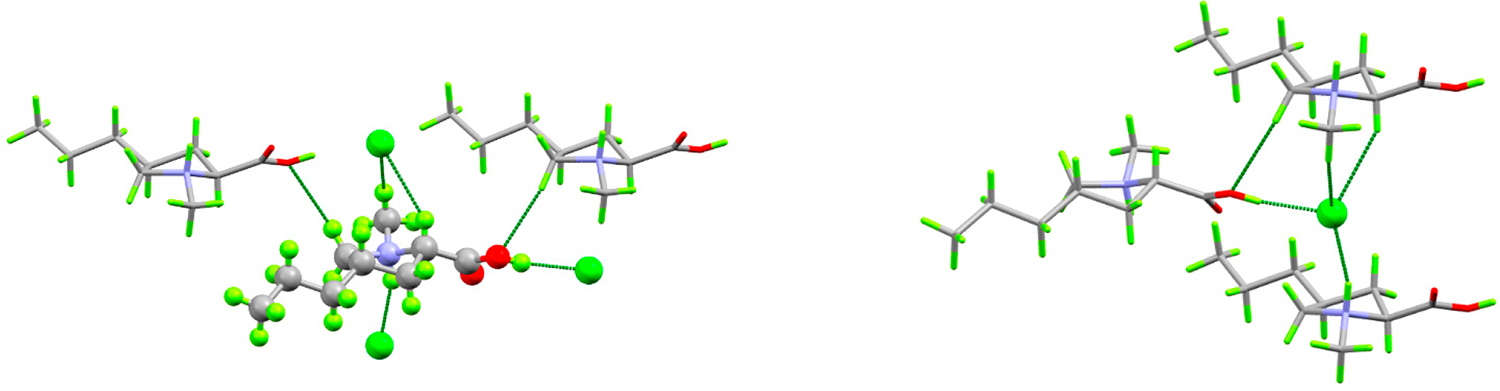
Packing plots showing short contact environments around the cation (**left**) and anion (**right**). Element colors are the same as in Figure 2. Dashed green lines indicate short contacts.

**Figure 4.**
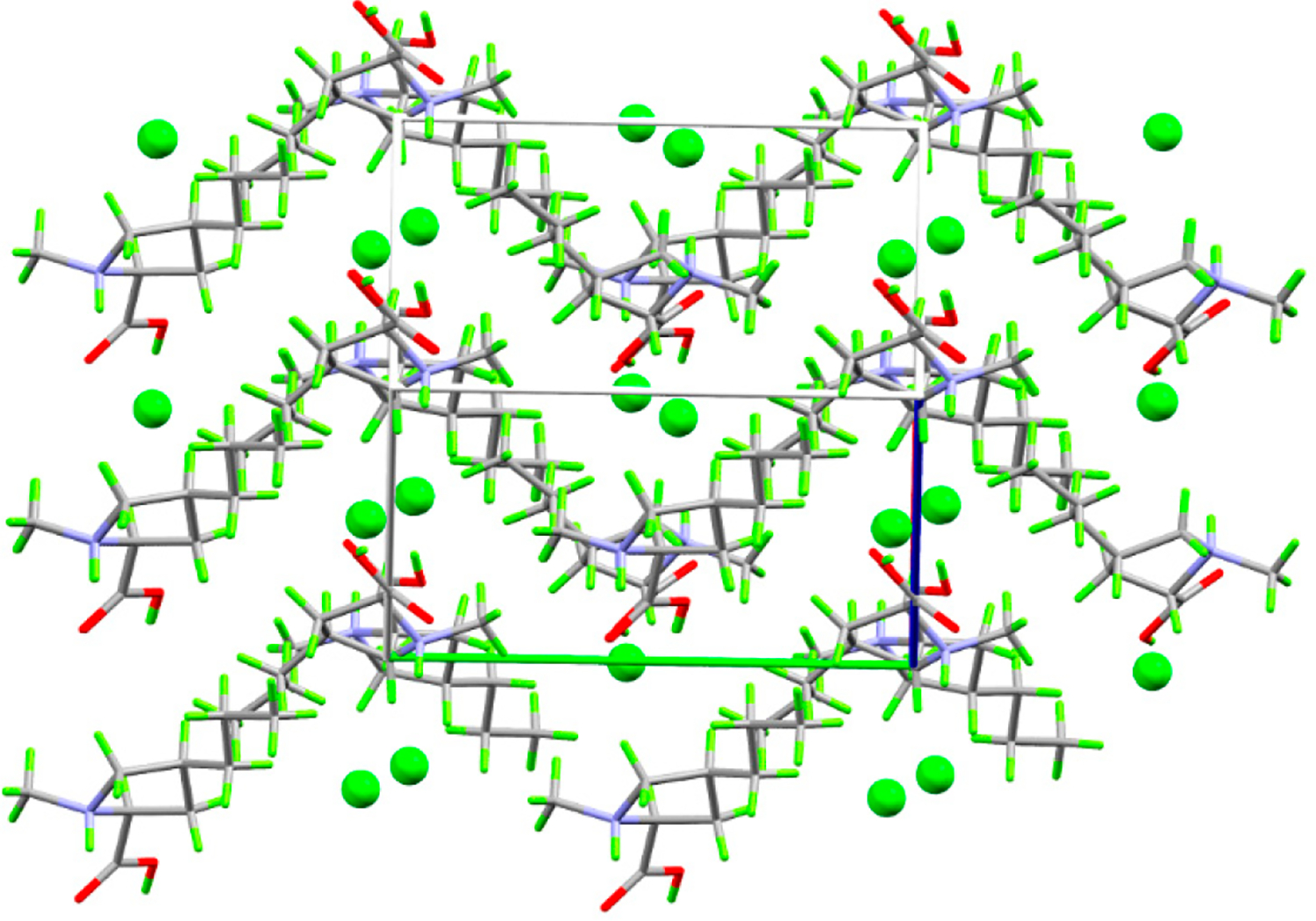
Packing plot showing 2 × 2 × 2 unit cells viewed down the *ac* face diagonal. Axes are color coded: *a* = red; *b* = green; *c* = blue.

The geometry of the cation is very similar to *N*-methyl-L-proline hydrochloride [21]. C-C distances around the ring range from 1.501(2) Å to 1.539(3) Å. Bond distances involving the substituted carbon atom are slightly longer than the corresponding distances in *N*-methyl-L-proline hydrochloride. Interior ring angles are distorted to acute values ranging from 101.93(2)° to 105.95(2)°, with the corresponding angles in agreement in both structures. The shortest cation–anion contacts in (*4S*)-1-methyl-4-propyl-L-proline hydrochloride are 2.957(2) Å for the OH⋯Cl^−^ donor–acceptor distance and 3.069(2) Å for the NH^+^⋯Cl^−^ donor–acceptor distance. These correspond to strong hydrogen bonds and are very similar to those found in *N*-methyl-L-proline hydrochloride. The propyl chain is in the equatorial position, and its carbon atoms adopt an all-*anti* configuration.

(*4S*)-1-methyl-4-propyl-L-proline hydrochloride crystallizes neatly in the orthorhombic space group P2_1_2_1_2_1_ with one formula unit per asymmetric unit. Each cation makes short (less than the sum of the van der Waals radii) contacts with three neighboring anions and two cations, while each anion makes short contact with three cations (Figure 3). The cation–anion contact includes two strong hydrogen bonds and a third in which the anion interacts with the CH_2_ group *alpha* to the ammonium center and on which the formal positive charge is delocalized. These positively charged CH_2_ groups also donate hydrogen bonds to the carboxylic acid OH oxygen atoms. The strong hydrogen bonds link the ions into infinite, charge-ordered, head-to-tail chains parallel to the *c* axis, while the electrostatic interactions between the *alpha* CH_2_ groups and chloride anions link these along the *a* axis to form infinite layers with the propyl groups projecting into the interlayer space (Figure 4).

A search of the Cambridge Structural Database [22] for 4-substituted proline derivatives (including those functionalized at the N and OH positions) resulted in 240 hits, of which 187 were unique structures where the substituents could be clearly assigned *cis* or *trans* with respect to the α-carbonyl group. Structures with *cis* substituents are significantly underrepresented, accounting for only 51 of these entries, and many of these studies emphasize differences between both epimers in aspects such as prochirality [23,24], metal coordination [25,26], noncovalent interactions in biomacromolecules [27–29], and access to other natural products [30,31] and pharmaceuticals [32,33].

## 3. Conclusions

In summary, we present a concise preparation of (*4S*)-1-methyl-4-propyl-L-proline, which commences from the widely available chiron (*cis*)-4-hydroxy-L-proline. Single-crystal X-ray diffraction analysis confirms the identity of the final target as its hydrochloride salt. We expect this work to be of interest to medicinal chemists engaged in the discovery of new antibacterial antibiotics and to synthetic chemists tasked with the preparation of unusual amino acids.

## Supplementary Material

SI

## Data Availability

The data underlying this study are available in the published article and its Supplementary Materials.
