# Supplementary material for: Stereoselective Preparation of (4S)-1-Methyl-4-Propyl-L-Proline Commencing from (cis)-4-Hydroxy-L-Proline
Source: Molbank. Author manuscript; Available in PMC 2026 Mar 7. (PMC12965518; doi:10.3390/M2003)
Supplement: SI [file NIHMS2079572-supplement-SI.pdf]

Supporting Information

Stereoselective Preparation of (*4S*)-1-Methyl-4-Propyl-L-Proline Commencing from (*cis*)-4-Hydroxy-L-Proline

Gour Hari Mandal,[a] Shifali Choudhary,[a] Steven P. Kelley,[b] and Shyam Sathyamoorthi[*, a]

[a] Department of Medicinal Chemistry, University of Kansas, Lawrence, Kansas 66047, United States

[b] Department of Chemistry, University of Missouri—Columbia, Columbia, Missouri 65211, United States

Table of Contents

I. General Considerations
II. Experimental Procedures, Tabulated Characterization Data, and NMR Spectra
III. X-ray Crystallographic Data

I. General Considerations

All reagents were obtained commercially unless otherwise noted. Solvents were purified by passage under 10 psi N_2 through activated alumina columns. Infrared (IR) spectra were recorded on a Thermo Scientific™ Nicolet™ iS™5 FT-IR Spectrometer; data are reported in frequency of absorption (cm^{-1}). ^1H NMR spectra were recorded at 400, 500, or 600 MHz. Data are recorded as: chemical shift in ppm referenced internally using residual solvent peaks, multiplicity (s = singlet, br s = broad singlet, d = doublet, t = triplet, q = quartet, m = multiplet or overlap of nonequivalent resonances, qdd = quartet of doublet of doublets, tdt = triplet of doublet of triplets, dtq = doublet of triplet of quartets, qd = quartet of doublets, tdq = triplet of doublet of quartets), integration, coupling constant (Hz). ^{13}C NMR spectra were recorded at 101 or 126 MHz. Exact mass spectra were recorded using an electrospray ion source (ESI) either in positive mode or negative mode and with a time-of-flight (TOF) analyzer on a Waters LCT PremierTM mass spectrometer and are given in m/z. Thin Layer Chromatography (TLC) was performed on pre-coated glass plates (Merck) and visualized either with a UV lamp (254 nm) or by dipping into a solution of $KMnO_4$–K_2CO_3 in water followed by heating. Flash chromatography was performed on silica gel (230-400 mesh) or Florisil (60-100 mesh). "Room temperature" refers to an ambient temperature of 23 – 25 °C.

II. Experimental Procedures, Tabulated Characterization Data, and NMR Spectra

Step 1

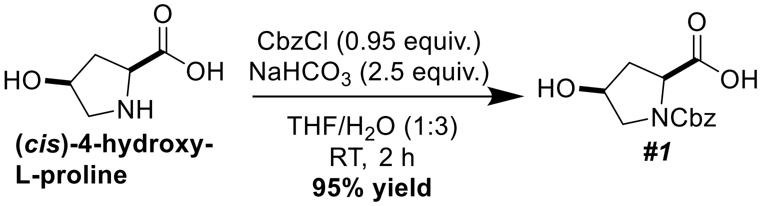

A 100 mL round bottom flask equipped with a magnetic stir bar was charged with *cis*-4-hydroxy-L-proline (3.0 g, 22.9 mmol, 1 equiv.), $NaHCO_3$ (4.8 g, 57.1 mmol, 2.5 equiv.) and H_2O (18.0 mL). Benzyl chloroformate (3.08 mL, 3.7 g, 21.7 mmol, 0.95 equiv.) in THF (6.0 mL) was added, and the mixture was stirred at room temperature for 2 h. Following this time, the reaction mixture was cooled to 0 °C using an ice-water bath, and then concentrated HCl was added dropwise until the solution reached a pH < 2. The mixture was transferred to a separatory funnel, and the aqueous layer was extracted with EtOAc (2 x 70 mL). The combined organic layers were washed with brine (30 mL), dried over Na_2SO_4, and filtered. The mixture was concentrated *in vacuo* to give compound **1** (5.78 g, 21.8 mmol, 95% yield) as a colorless, sticky gum.

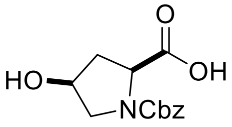

(2*S*,4*S*)-1-((benzyloxy)carbonyl)-4-hydroxypyrrolidine-2-carboxylic acid

Compound 1: *Note: Mixture of Rotamers*

^{1}H NMR (500 MHz, CD_3OD) δ 7.47 – 7.17 (m, 5H), 5.22 – 5.06 (m, 2H), 4.46 – 4.33 (m, 2H), 3.68 (dt, J = 10.7, 5.0 Hz, 1H), 3.47 – 3.39 (m, 1H), 2.43 (dddd, J = 20.3, 13.8, 9.3, 5.1 Hz, 1H), 2.12 (ddq, J = 13.2, 6.6, 3.1 Hz, 1H).

^{13}C{^{1}H} NMR (126 MHz, CD_3OD) δ 176.0, 175.7, 156.8, 156.5, 138.0, 137.9, 129.5, 129.4, 129.1, 129.0, 128.9, 128.6, 70.8, 70.0, 68.28, 68.25, 59.3, 59.1, 55.7, 55.4, 39.8, 39.0.

IR ν 3035, 2965, 1755, 1647, 1436, 1010 cm^{-1}.

HRMS (ESI) m/z = $[M + Na]^+$ Calcd $C_{13}H_{15}NO_5Na^+$ 288.0848. Found 288.0821 (9.4 ppm error).

$[\alpha]_D^{21.5}$ = -33.38 (c = 1.6 g/100 mL, MeOH).

Compound 1 (CD$_3$OD, ^{1}H NMR: 500 MHz, ^{13}C{^{1}H} NMR: 126 MHz)

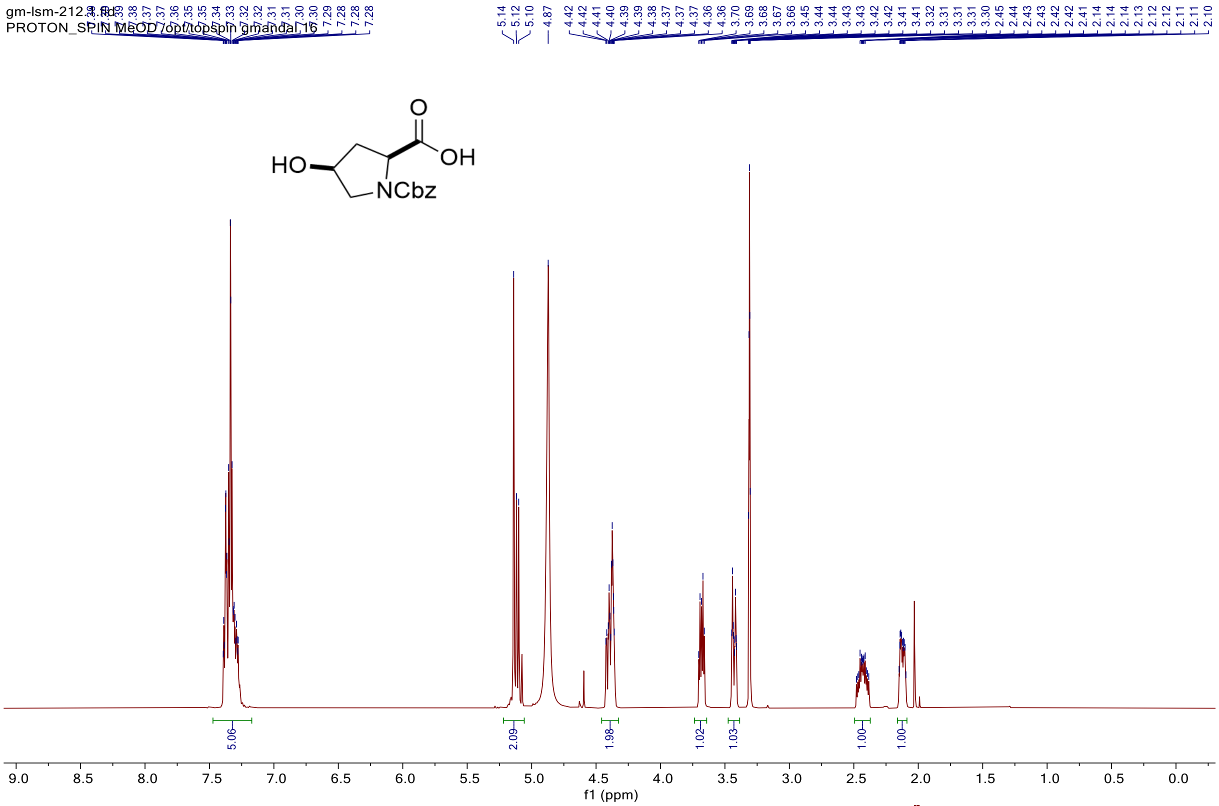

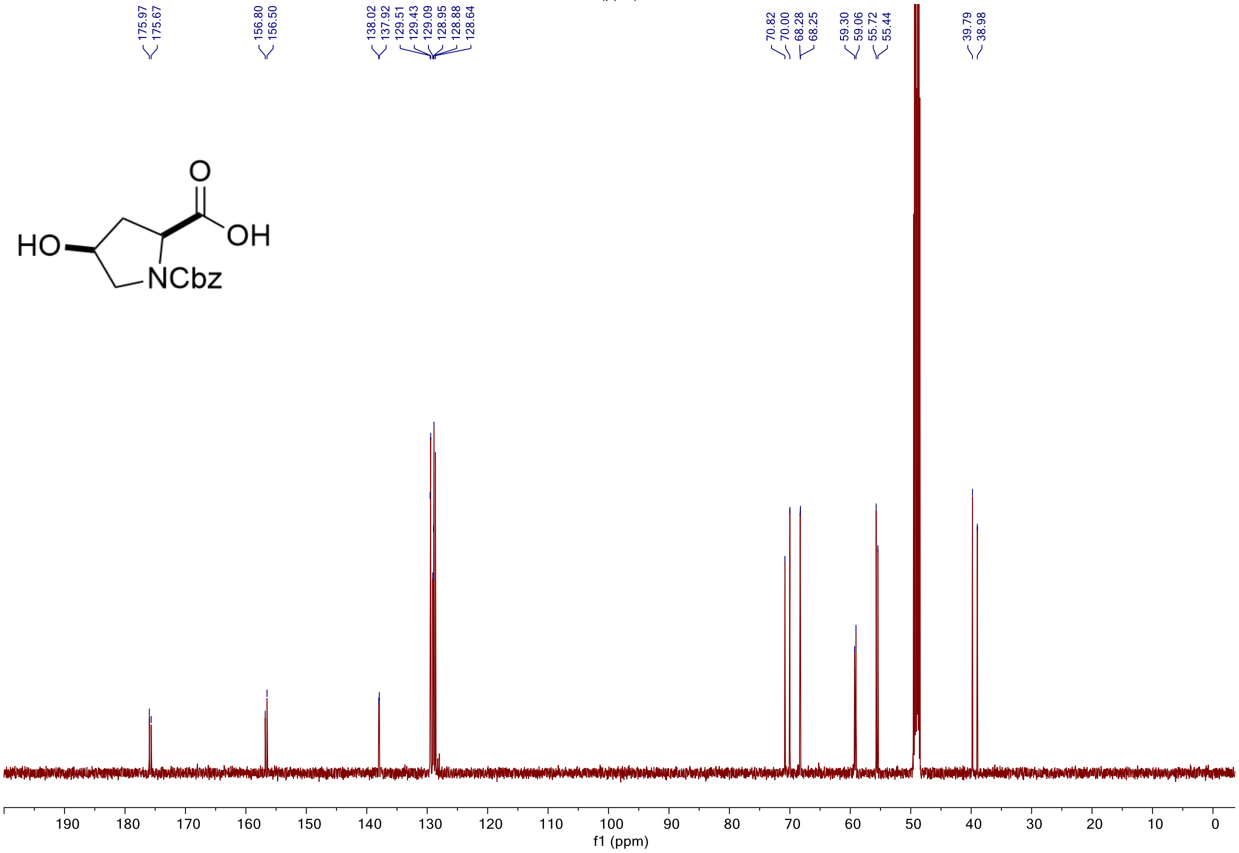

IR

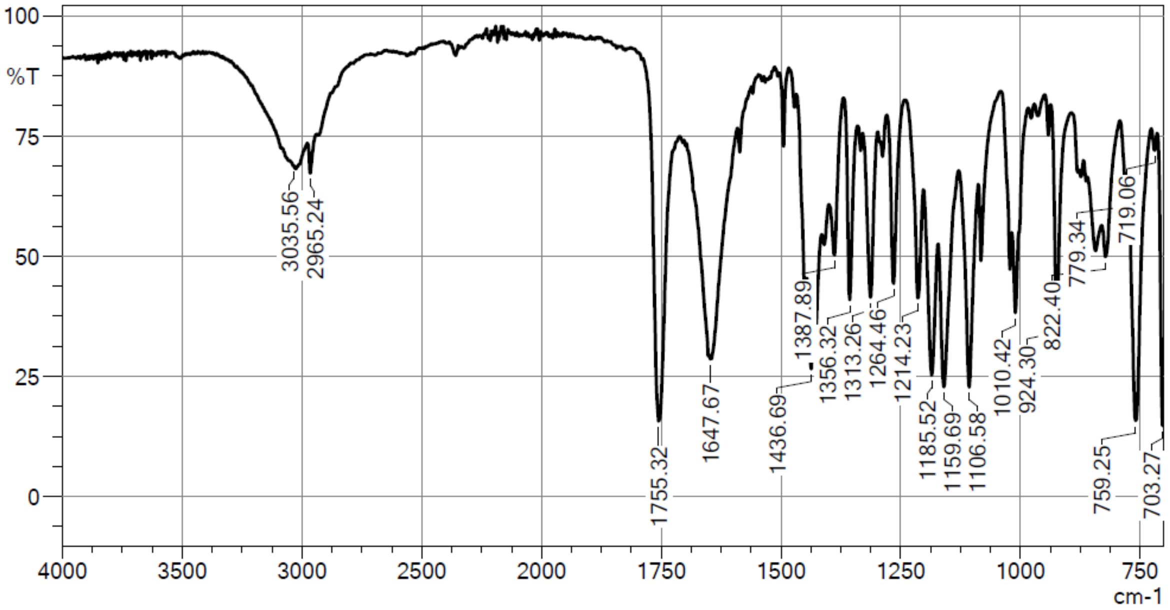

100
%T
75
50
25
0
3035.56
2965.24
1755.32
1647.67
1436.69
1387.89
1356.32
1313.26
1264.46
1214.23
1185.52
1159.69
1106.58
1010.42
924.30
822.40
779.34
759.25
719.06
703.27
4000
3500
3000
2500
2000
1750
1500
1250
1000
750
cm-1

HRMS

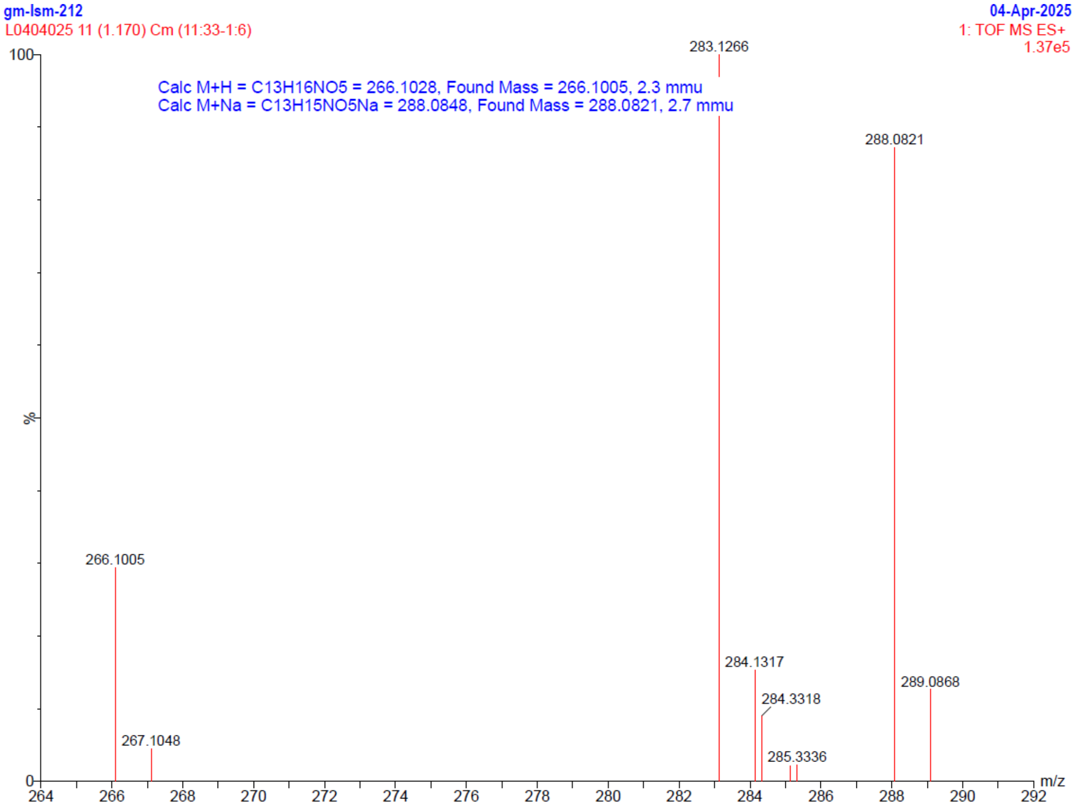

gm-lsm-212
L0404025 11 (1.170) Cm (11:33-1:6)
04-Apr-2025
1: TOF MS ES+
1.37e5
Calc M+H = C13H16NO5 = 266.1028, Found Mass = 266.1005, 2.3 mmu
Calc M+Na = C13H15NO5Na = 288.0848, Found Mass = 288.0821, 2.7 mmu
283.1266
288.0821
266.1005
267.1048
284.1317
284.3318
285.3336
289.0868
100
%
0
m/z

Step 2

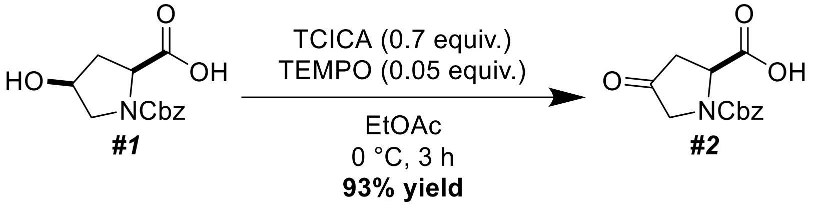

A 100 mL round bottom flask equipped with a magnetic stir bar was charged with compound **1** (5.88 g, 22.2 mmol, 1 equiv.) and EtOAc (40.0 mL). The mixture was cooled to 0 °C using an ice-water bath. TEMPO (173 mg, 1.1 mmol, 0.05 equiv.) and trichloroisocyanuric acid (3.61 g, 15.5 mmol, 0.7 equiv.) were added. The mixture was stirred at 0 °C for 3 h. Following this time, the reaction mixture was filtered to remove insoluble material. The filtrate was cooled to 0 °C using an ice-water bath and quenched by addition of saturated, aqueous $Na_2S_2O_3$ solution (10 mL) (**Caution! Exothermic**). The mixture was transferred to a separatory funnel and was extracted with EtOAc (2 x 70 mL). The combined organic layers were washed with brine (30 mL), dried over Na_2SO_4, filtered, and concentrated under reduced pressure to give a crude residue. This crude residue was then suspended in 100 mL of 10% EtOAc/hexanes, and this mixture was magnetically stirred for 2 h. The solid was recovered by filtration and washed with another 100 mL of 10% EtOAc/hexanes. The solid residue was then dried under high vacuum to yield compound **2** (5.44 g, 20.66 mmol, 93% yield) as a white powder.

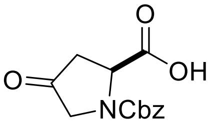

(*S*)-1-((benzyloxy)carbonyl)-4-oxopyrrolidine-2-carboxylic acid

Compound 2: *Note: Mixture of rotamers*

^{1}H NMR (500 MHz, $CDCl_3$) δ 10.75 (broad s, 1H), 7.36 – 7.18 (m, 5H), 5.35 – 4.94 (m, 2H), 4.80 (ddd, *J* = 20.2, 10.7, 2.6 Hz, 1H), 4.06 – 3.65 (m, 2H), 2.90 (td, *J* = 20.1, 10.6 Hz, 1H), 2.79 – 2.54 (m, 1H).

^{13}C{^{1}H} NMR (126 MHz, $CDCl_3$) *δ* 207.4, 207.0, 176.3, 175.7, 155.5, 154.4, 135.8, 135.7, 128.72, 128.67, 128.55, 128.46, 128.2, 128.1, 68.3, 68.1, 55.9, 52.6, 52.5, 41.1, 40.3.

IR *ν* 3676, 2966, 1755, 1644, 1437, 1010 cm^{-1}.

HRMS (ESI) m/z = $[M + Na]^+$ Calcd $C_{13}H_{13}NO_5Na^+$ 286.0691. Found 286.0679 (4.2 ppm error).

$[\alpha]_D^{22.1}$ = -4.35 (*c* 1.33 g/100 mL, MeOH).

The ^{1}H NMR shifts of this compound are reported in *Organic Letters*, **2022**, *24*, 3421 – 3425, and our data matches.

Compound 2 ($CDCl_3$, ^1H NMR: 500 MHz, $^{13}C\{^1H\}$ NMR: 126 MHz)

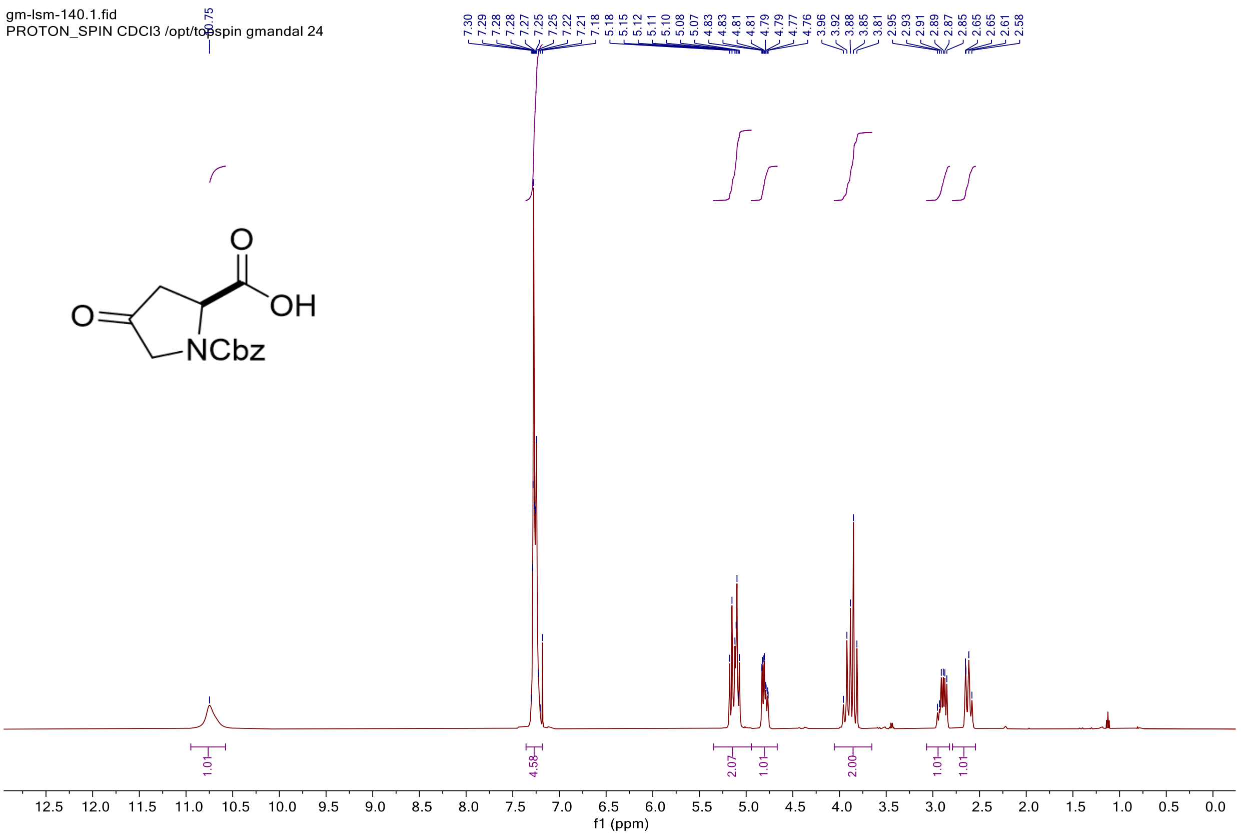

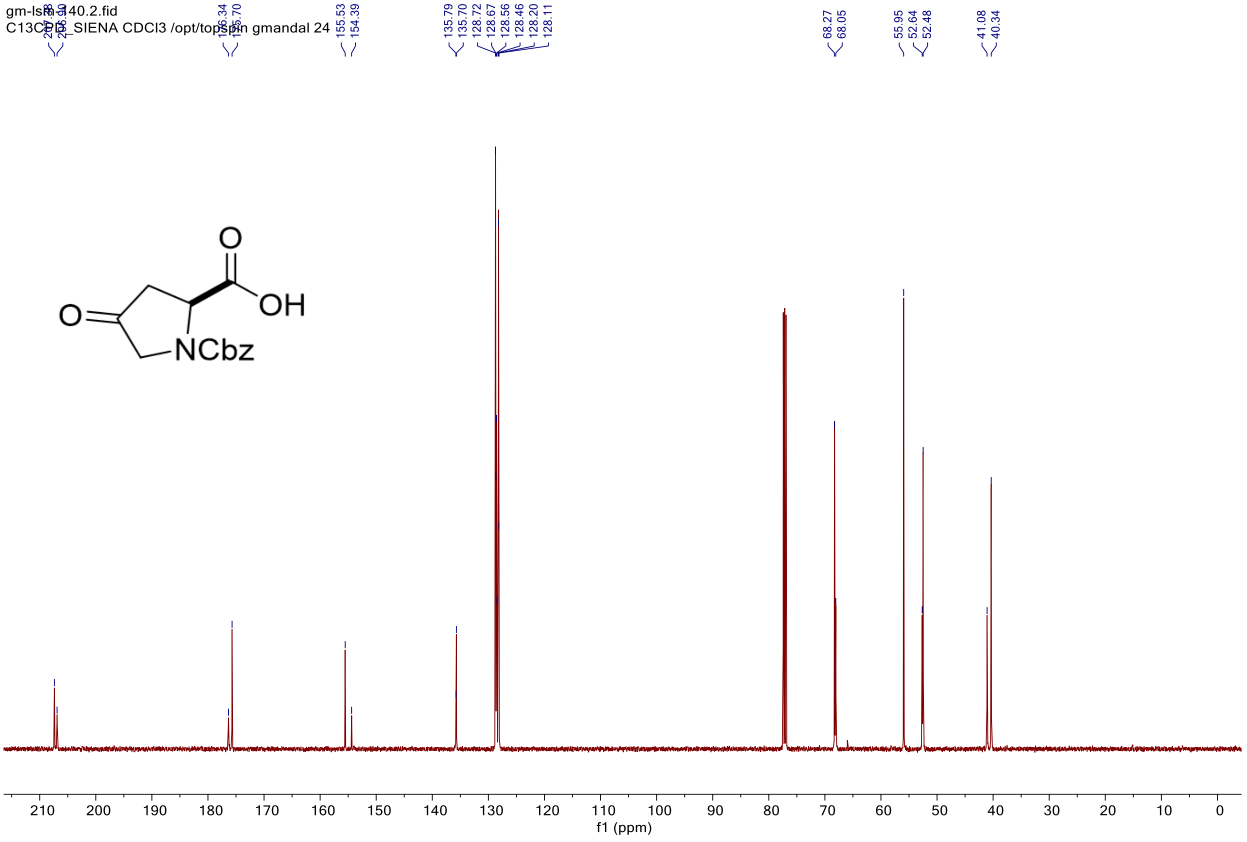

IR

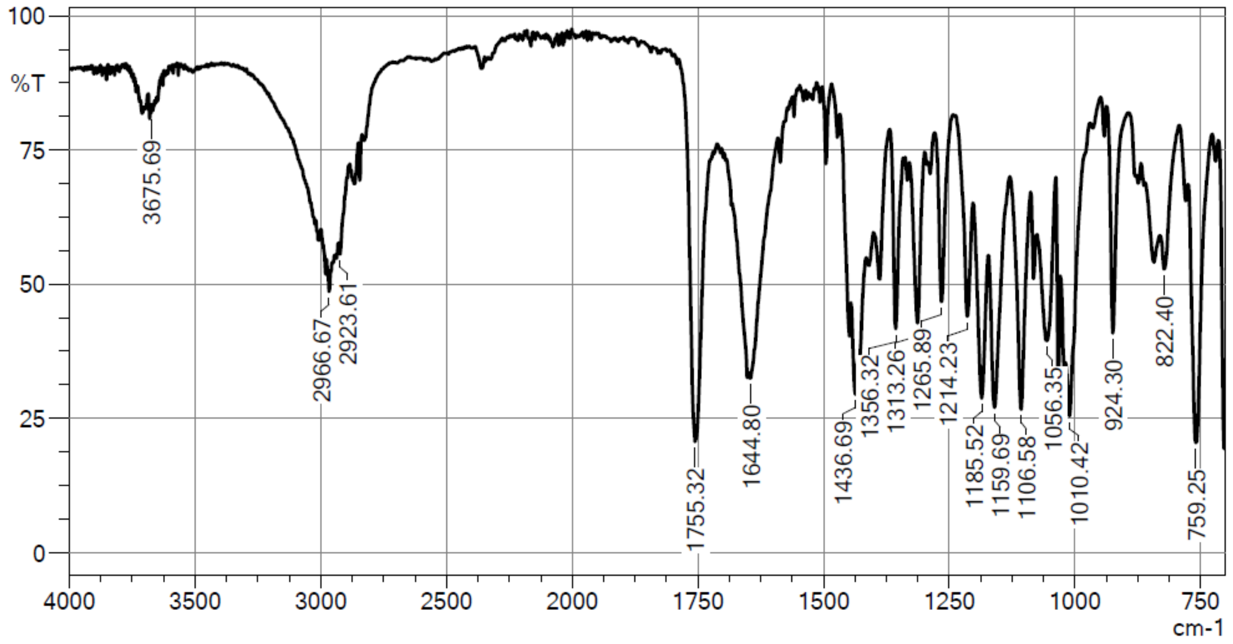
%T
3675.69
2966.67
2923.61
1755.32
1644.80
1436.69
1356.32
1313.26
1265.89
1214.23
1185.52
1159.69
1106.58
1056.35
1010.42
924.30
822.40
759.25
cm-1

HRMS

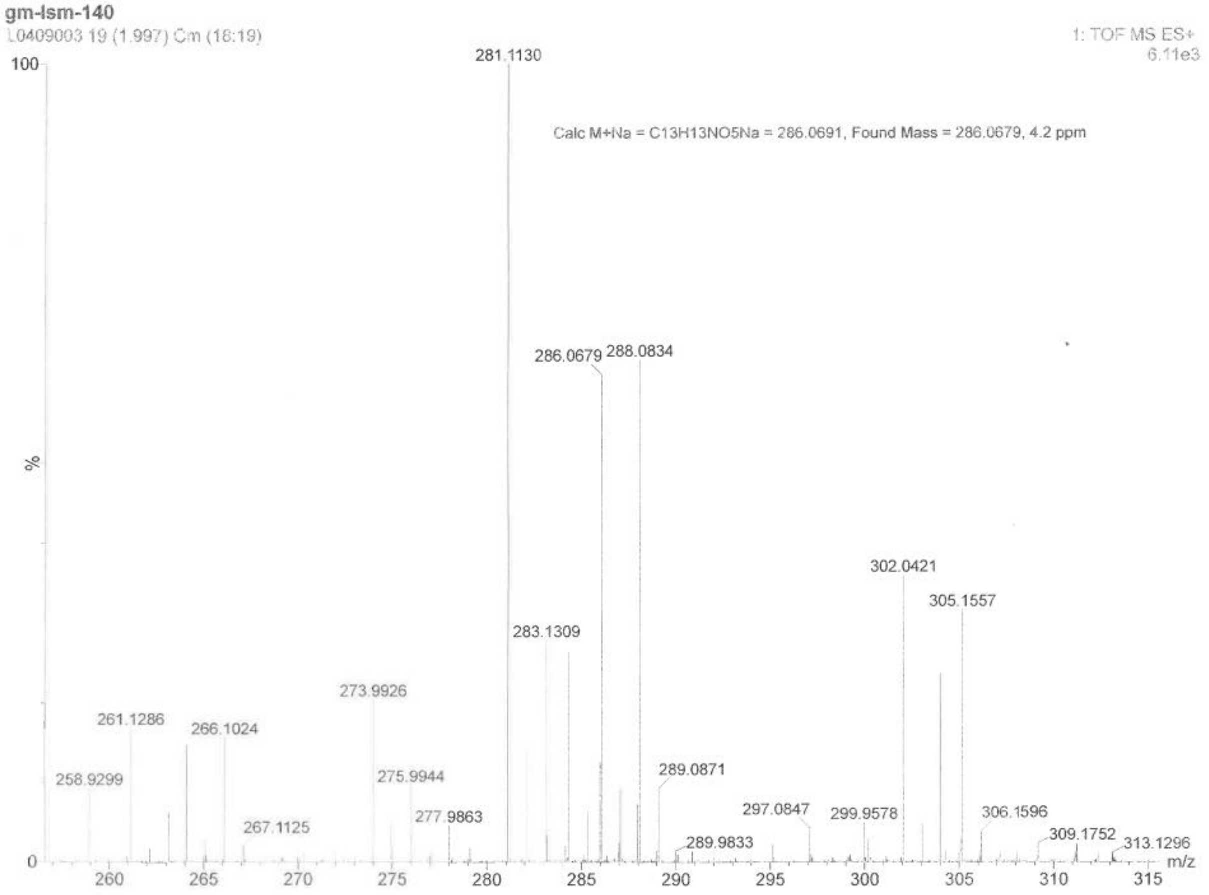
gm-lsm-140
L0409003 19 (1.997) Cm (18:19)
1: TOF MS ES+
6.11e3
Calc M+Na = C13H13NO5Na = 286.0691, Found Mass = 286.0679, 4.2 ppm
281.1130
286.0679
288.0834
302.0421
305.1557
283.1309
273.9926
261.1286
266.1024
258.9299
275.9944
289.0871
277.9863
267.1125
297.0847
299.9578
306.1596
309.1752
313.1296
289.9833
%
m/z

Step 3

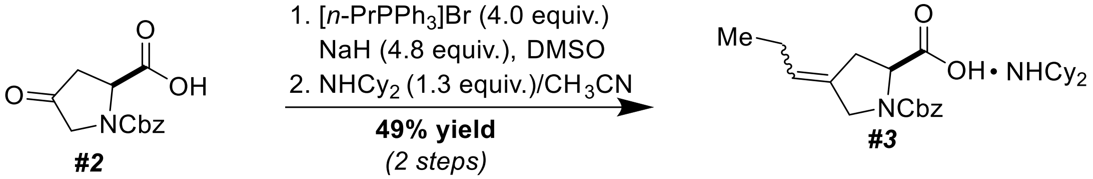

A 250 mL round-bottom flask equipped with a magnetic stir bar was charged with sodium hydride (0.875 g, 36.5 mmol, 4.8 equiv.) and anhydrous dimethyl sulfoxide (30 mL). With stirring, the solution was warmed to ~75 °C using an oil bath for 1 hour (**Caution-Exotherm!**). After cooling to room temperature, propyltriphenylphosphonium bromide (11.7 g, 30.4 mmol, 4.0 equiv.) was added in one bolus. The resulting orange solution was stirred for 1 hour to ensure complete ylide formation. A solution of ketone **2** (2 g, 7.6 mmol, 1 equiv.) in 4 ml of dimethyl sulfoxide was added over a period of 10 min. The resulting mixture was stirred for 30 min at room temperature and then at 70 °C (warmed using an oil bath) for 4 hours. The reaction mixture was cooled to room temperature and then to 0 °C using an ice-water bath. The mixture was then treated with 5% (w/w) aqueous potassium bicarbonate solution (50 mL) and further diluted with water (50 mL). The mixture was filtered to remove insoluble material, and the filtrate was transferred to a separatory funnel and washed with diethyl ether (2 x 100 mL). The ether wash was discarded. The aqueous layer was transferred to an Erlenmeyer flask equipped with a magnetic stir bar, cooled to ~10 °C using an ice-water bath, and, with vigorous stirring, was acidified by dropwise addition of 1 M aqueous HCl solution to a pH of approximately 2. The solution was then transferred to a separatory funnel and was extracted with diethyl ether (3 x 100 mL). The combined organic extracts were washed with saturated, aqueous $NaHSO_3$ solution (2 x 30 mL) and H_2O (50 mL). After drying over $MgSO_4$ and filtering, the solvent was removed by evaporation under reduced pressure. The resulting residue was dissolved in CH_3CN (10 mL), transferred to a 50 mL round-bottom flask, and swirled with dicyclohexylamine (2.0 ml, 1.82 g, 10.0 mmol, 1.3 equiv.). After 15 minutes, the solution was concentrated under reduced pressure, giving a gray solid. This solid was washed with diethyl ether (2 x 40 mL) and was dried under high vacuum to give **3** (1.75 g, 3.72 mmol, 49% yield) as a mixture of E/Z isomers.

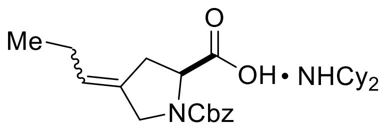

dicyclohexylamine (*S*)-1-((benzyloxy)carbonyl)-4-propylidenepyrrolidine-2-carboxylate

Compound 3: *Note: Mixture of E/Z isomers and rotamers*

^{1}H NMR (400 MHz, CD_3OD) δ 7.52 – 7.17 (m, 5H), 5.44 – 5.22 (m, 1H), 5.21 – 5.00 (m, 2H), 4.46 – 4.26 (m, 1H), 4.23 – 4.11 (m, 1H), 4.10 – 3.98 (m, 1H), 3.23 – 3.05 (m, 2H), 3.01 – 2.77 (m, 1H), 2.75 – 2.55 (m, 1H), 2.13 – 1.93 (m, 6H), 1.92 – 1.76 (m, 4H), 1.75 – 1.61 (m, 2H), 1.47 – 1.27 (m, 8H), 1.26 – 1.10 (m, 2H), 1.06 – 0.85 (m, 3H).

^{13}C{^{1}H} NMR (101 MHz, CD_3OD) δ 179.7, 179.5, 157.1, 156.9, 156.8, 138.4, 138.3, 135.9, 135.1, 129.5, 129.4, 128.9, 128.8, 128.7, 128.4, 125.18, 125.15, 124.9, 124.8, 67.89, 67.85, 62.9, 62.7, 62.4, 62.3, 54.42, 54.39, 52.4, 52.0, 38.5, 37.8, 34.3, 33.6, 30.59, 30.55, 26.2, 25.5, 23.7, 23.6, 23.4, 14.3, 14.2.

IR ν 3394, 2939, 2800, 1690, 1596, 1019 cm^{-1}.

HRMS (ESI) m/z = $[M + Na]^+$ Calcd $C_{16}H_{19}NO_4Na^+$ 312.1212. Found 312.1200 (3.8 ppm error).

Compound 3 (CD_3OD, ^{1}H NMR: 400 MHz, $^{13}C\{^{1}H\}$ NMR: 101 MHz)

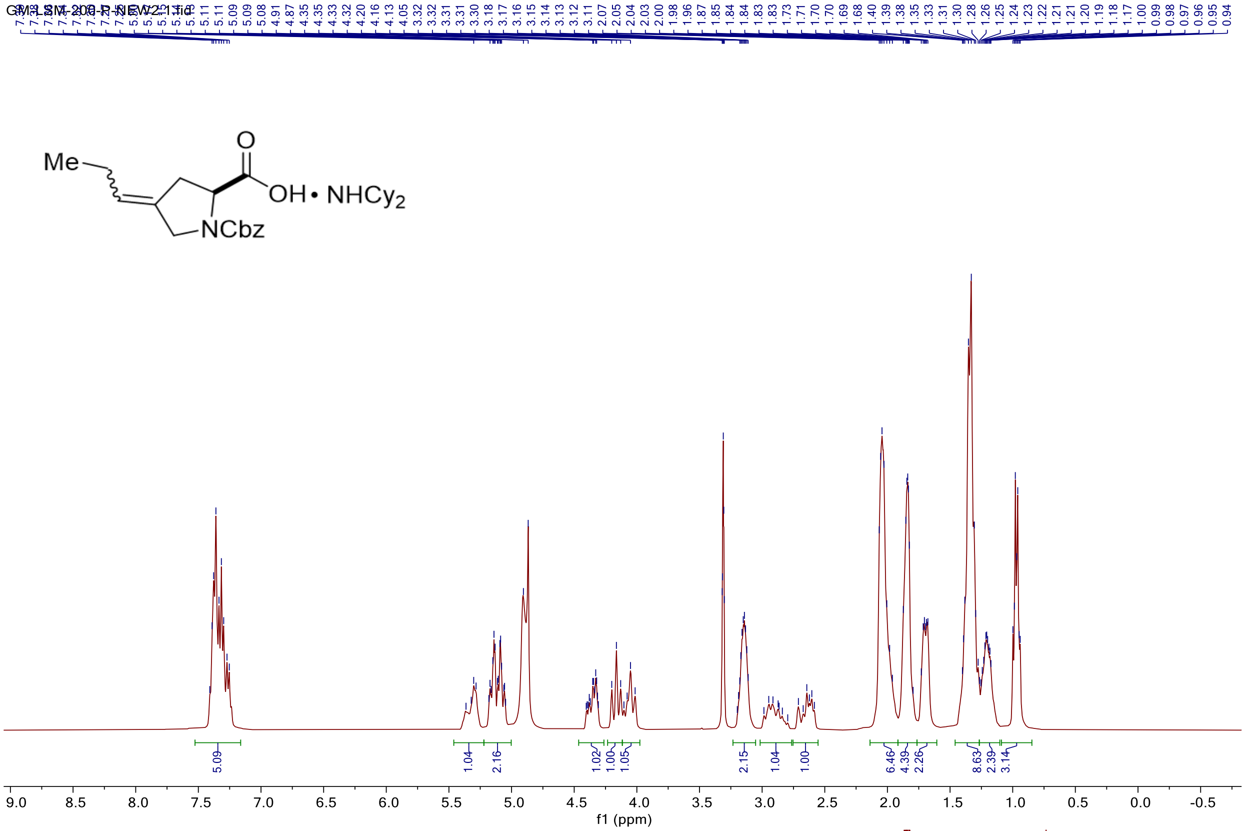

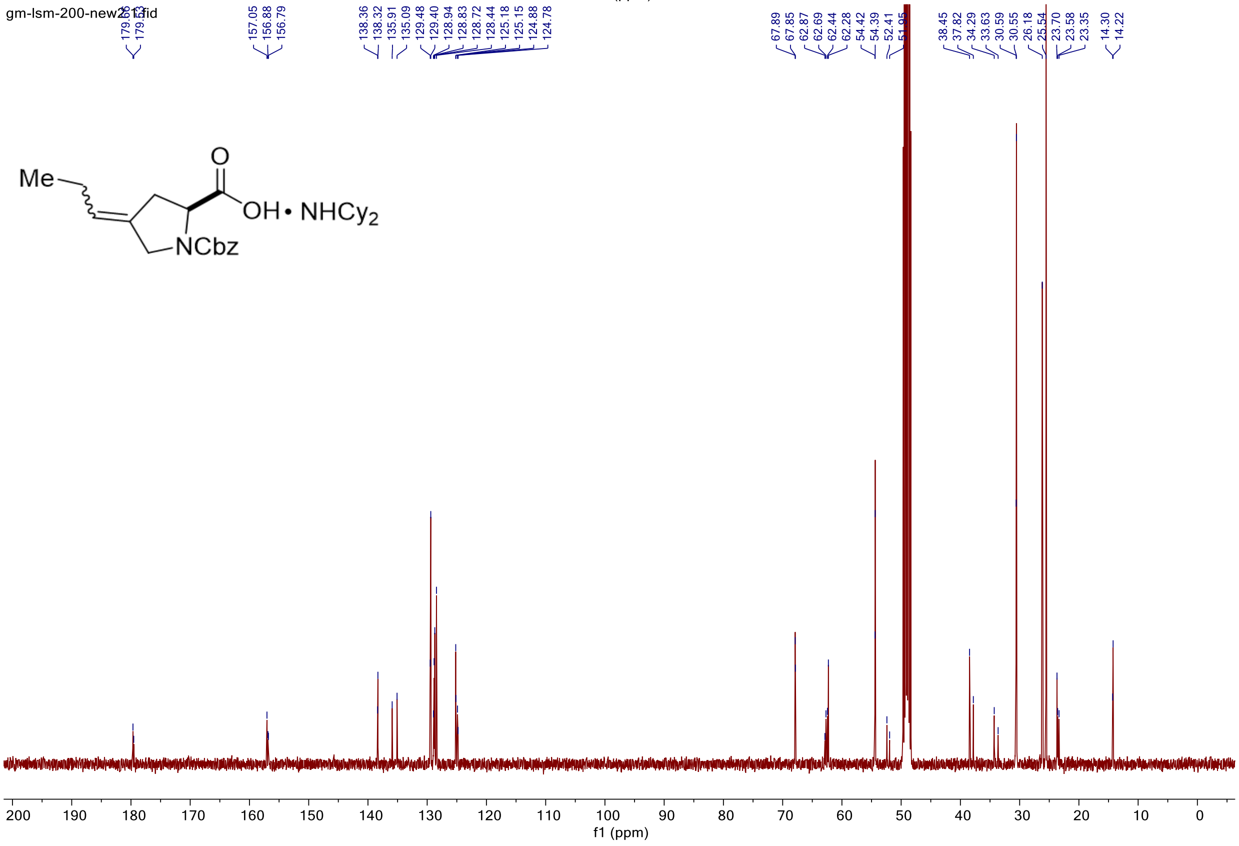

IR

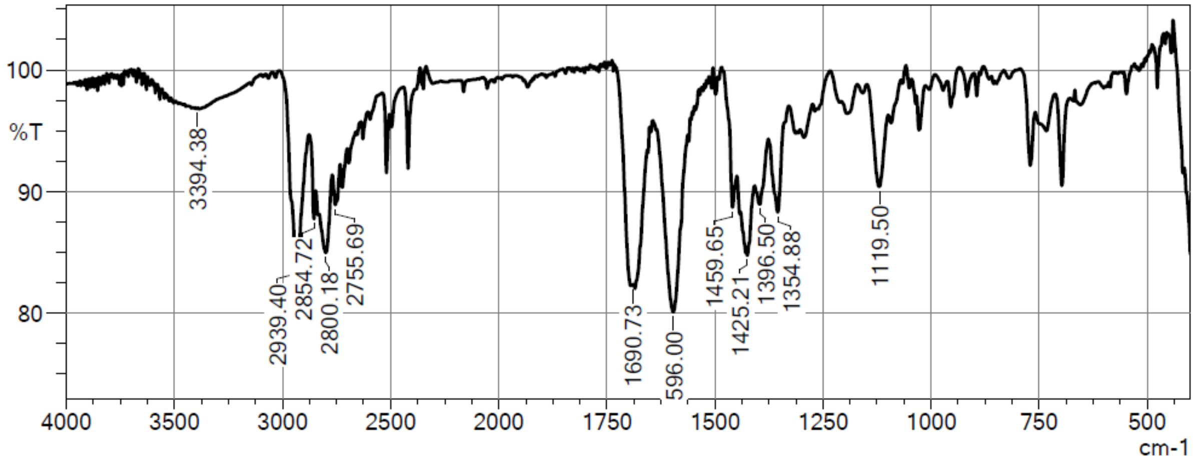
100
%T
90
80
3394.38
2939.40
2854.72
2800.18
2755.69
1690.73
596.00
1459.65
1425.21
1396.50
1354.88
1119.50
4000
3500
3000
2500
2000
1750
1500
1250
1000
750
500
cm-1

HRMS

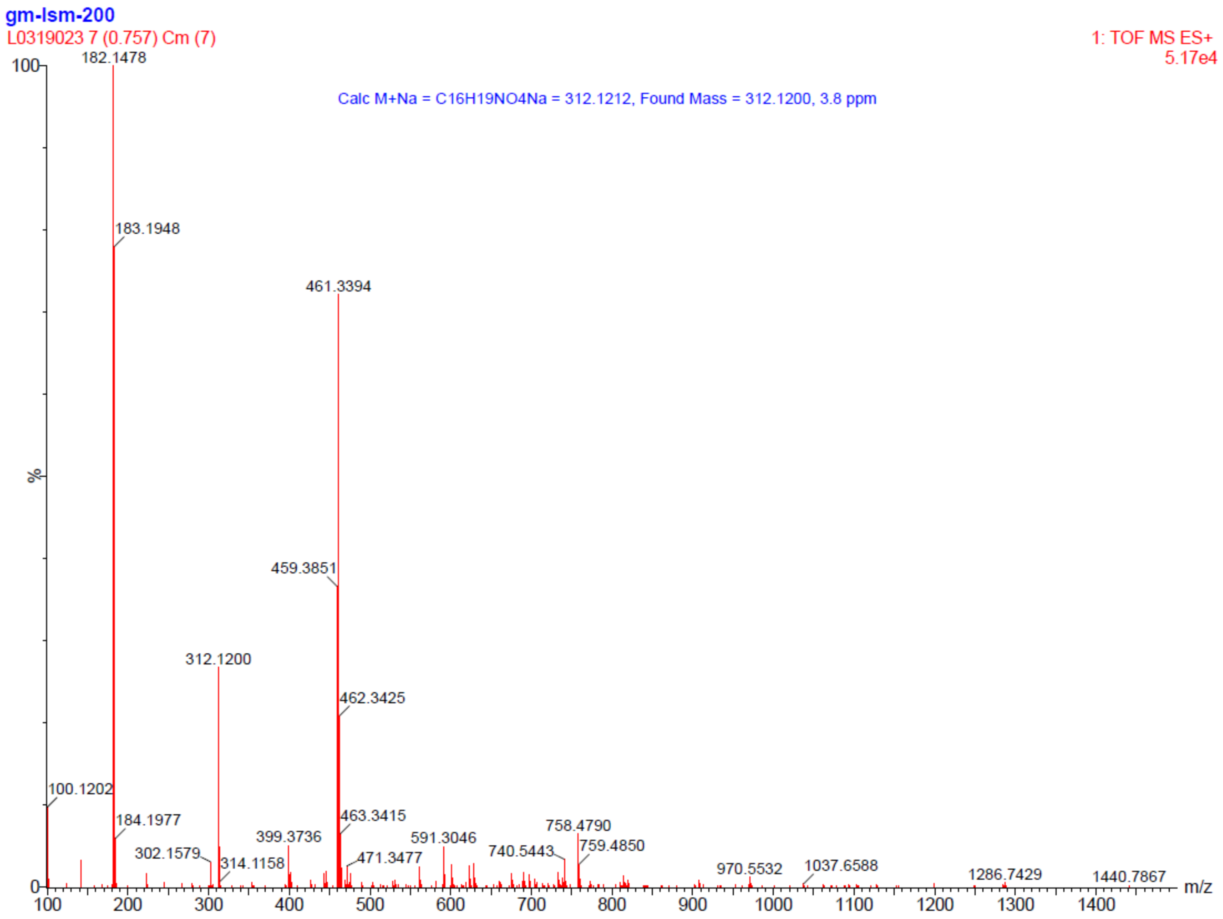
gm-lsm-200
L0319023 7 (0.757) Cm (7)
1: TOF MS ES+
5.17e4
Calc M+Na = C16H19NO4Na = 312.1212, Found Mass = 312.1200, 3.8 ppm
100
%
0
182.1478
183.1948
461.3394
459.3851
312.1200
462.3425
100.1202
184.1977
463.3415
399.3736
591.3046
758.4790
740.5443
759.4850
302.1579
314.1158
471.3477
970.5532
1037.6588
1286.7429
1440.7867
100
200
300
400
500
600
700
800
900
1000
1100
1200
1300
1400
m/z

Step 4

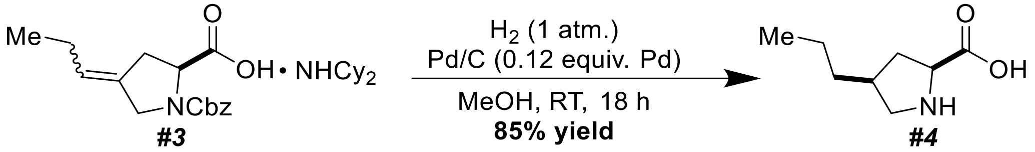

A 50 mL round-bottom flask equipped with a magnetic stir bar was charged with compound **3** (1.68 g, 3.57 mmol, 1 equiv.), 10% Pd/C (450 mg of mixture, 45 mg of Pd, 0.423 mmol of Pd, ~0.12 equiv.), and MeOH (25 mL). N_2 gas was bubbled through the solution for 15 min. A balloon of H_2 gas was affixed to the flask, and the reaction was stirred under ~1 atm of H_2 for 18 hours at room temperature. Following this time, the mixture was filtered through a short plug of Celite using MeOH (50 mL). The filtrate was concentrated under reduced pressure. The resulting brown solid was washed with diethyl ether (3 x 30 mL) and was then dried under high vacuum to give compound **4** (0.48 g, 3.05 mmol, 85% yield) as a light brown solid.

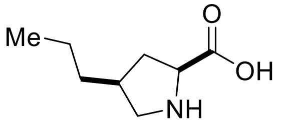

(2*S*,4*S*)-4-propylpyrrolidine-2-carboxylic acid

Compound 4:

^{1}H NMR (500 MHz, CD_3OD) δ 4.03 – 3.90 (m, 1H), 3.40 (dd, J = 11.4, 7.3 Hz, 1H), 2.91 (dd, J = 11.4, 9.8 Hz, 1H), 2.53 (dddd, J = 13.0, 8.0, 6.8, 0.9 Hz, 1H), 2.31 (dqd, J = 9.8, 7.0, 2.8 Hz, 1H), 1.63 (dt, J = 13.0, 9.6 Hz, 1H), 1.52 – 1.30 (m, 4H), 0.94 (t, J = 7.1 Hz, 3H).

^{13}C{^{1}H} NMR (126 MHz, CD_3OD) δ 174.1, 62.8, 51.6, 40.1, 36.6, 35.6, 22.3, 14.3.

IR v 3247, 2952, 1610, 1374, 820 cm^{-1}.

HRMS (ESI) m/z = $[M + H]^+$ Calcd $C_8H_{16}NO_2^+$ 158.1181. Found 158.1182 (0.6 ppm error).

$[\alpha]_D^{24.4}$ = -57.4 (c 1.0 g/100 mL, H_2O).

Compound 4 (CD_3OD, ^{1}H NMR: 500 MHz, $^{13}C\{^{1}H\}$ NMR: 126 MHz)

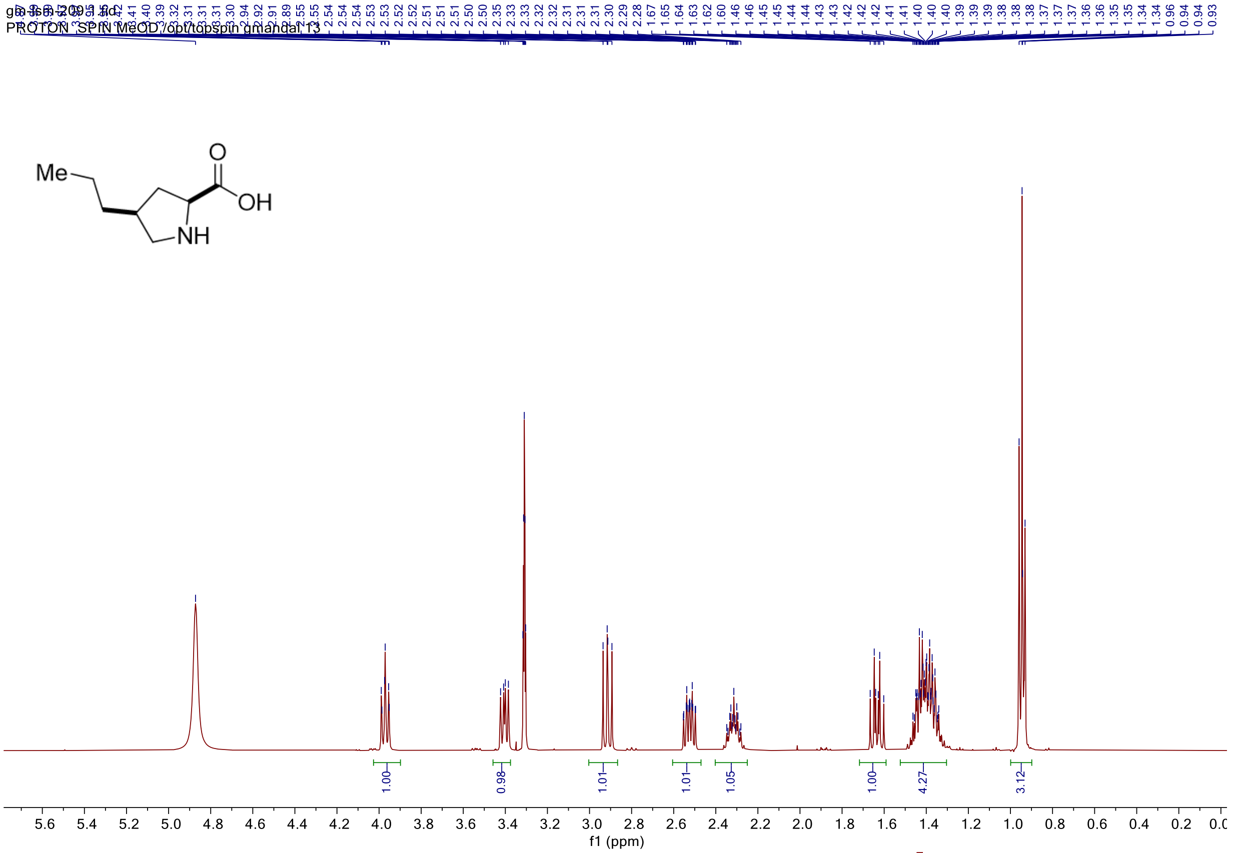

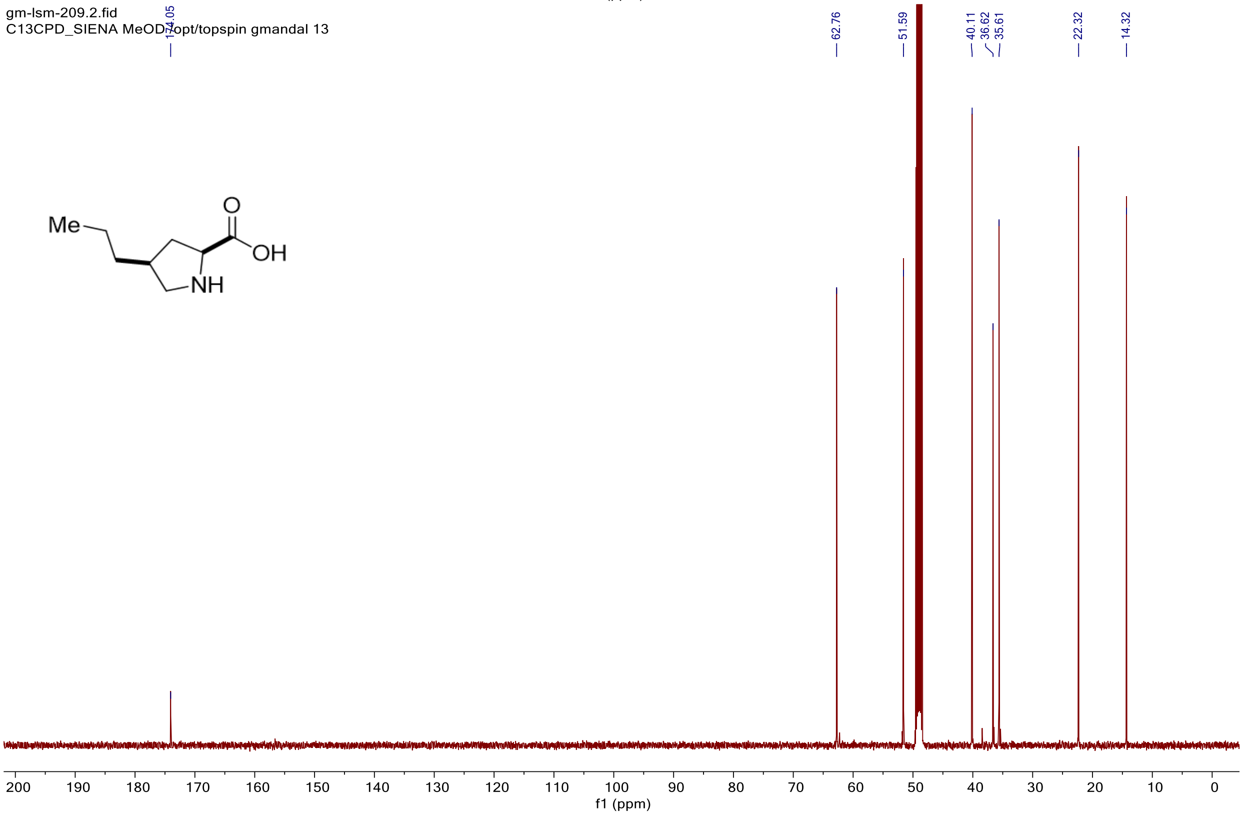

IR

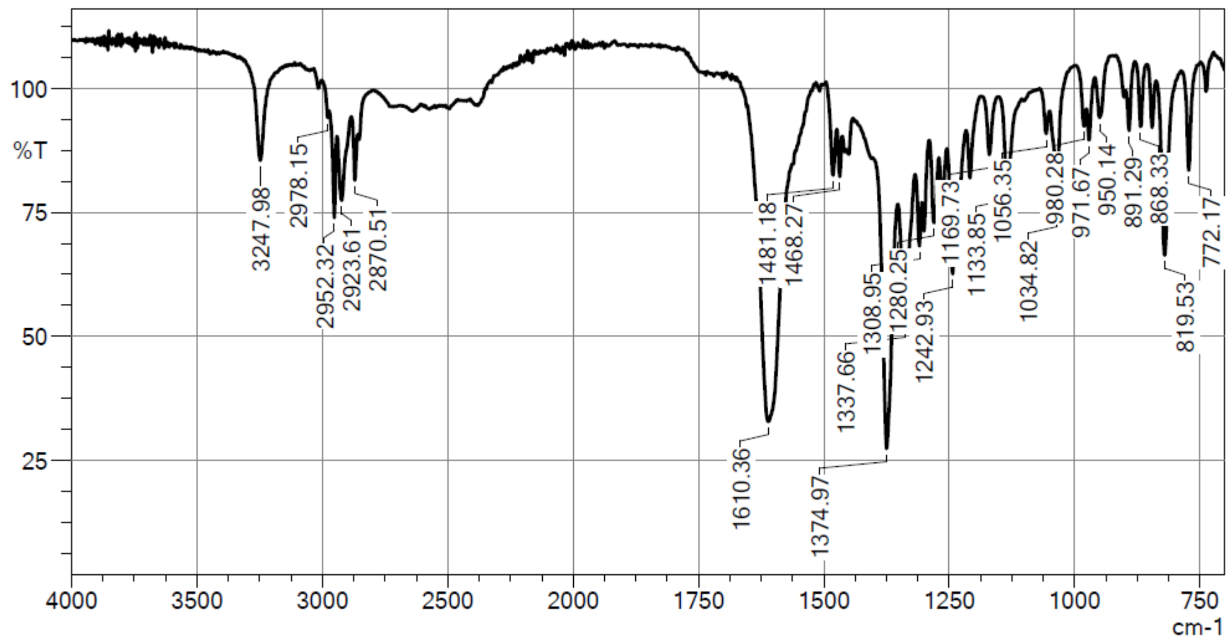

100
%T
75
50
25
3247.98
2978.15
2952.32
2923.61
2870.51
1610.36
1481.18
1468.27
1374.97
1337.66
1308.95
1280.25
1242.93
1169.73
1133.85
1056.35
1034.82
980.28
971.67
950.14
891.29
868.33
819.53
772.17
4000
3500
3000
2500
2000
1750
1500
1250
1000
750
cm-1

HRMS

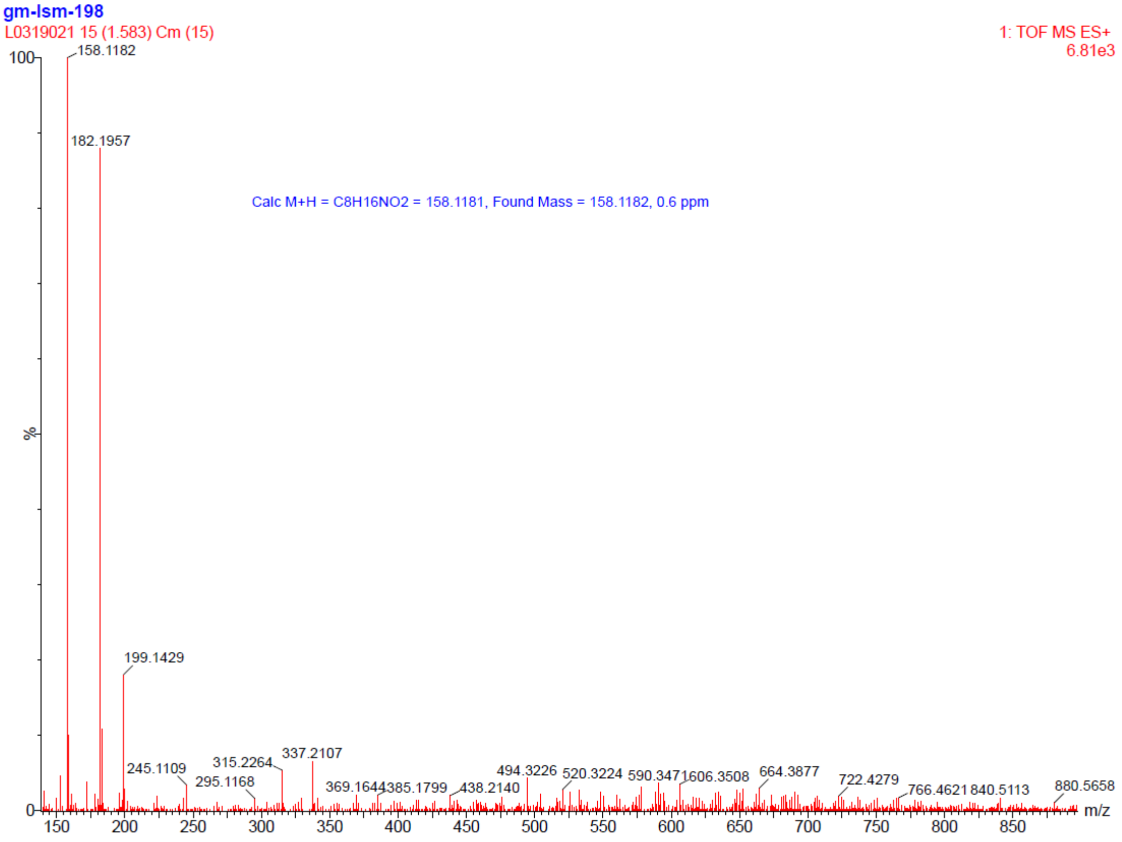

gm-lsm-198
L0319021 15 (1.583) Cm (15)
1: TOF MS ES+
6.81e3
Calc M+H = C8H16NO2 = 158.1181, Found Mass = 158.1182, 0.6 ppm
158.1182
182.1957
199.1429
245.1109
295.1168
315.2264
337.2107
369.1644
385.1799
438.2140
494.3226
520.3224
590.3471
606.3508
664.3877
722.4279
766.4621
840.5113
880.5658
%
m/z

Step 5

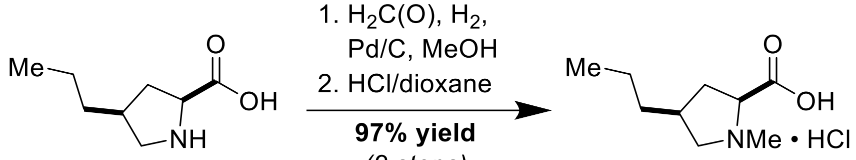

#4

(*4S*)-1-methyl-4-propyl-L-proline *(hydrochloride salt)*

A 50 mL round-bottom flask equipped with a magnetic stir bar was charged with compound **4** (0.475 g, 3.02 mmol, 1 equiv.), formaldehyde (37 wt% solution in water, 2 mL, 2.18 g of solution, 0.807 g of formaldehyde, 26.9 mmol, 8.9 equiv.), 10% Pd/C (120 mg of mixture, 12 mg of Pd, 0.113 mmol of Pd, ~0.04 equiv.), and MeOH (20 mL). N_2 gas was bubbled through the solution for 15 min. A balloon of H_2 gas was affixed to the flask, and the reaction was stirred under ~1 atm of H_2 for 16 hours at room temperature. Following this time, the mixture was filtered through a short plug of Celite using MeOH (50 mL). The filtrate was concentrated under reduced pressure. The crude residue was dissolved in dioxane (10 mL) and transferred to a 25 mL round-bottom flask equipped with a magnetic stir bar. 4 M HCl/dioxane solution (3 mL, 12 mmol of HCl, 4 equiv.) was added, and the solution was stirred for 10 min. The solvent was removed under reduced pressure, and the resulting brown solid was washed with diethyl ether (3 x 30 mL). The solid was dried under high vacuum to give (*4S*)-1-methyl-4-propyl-L-proline hydrochloride (0.61 g, 2.93 mmol, 97% yield) as a brown solid.

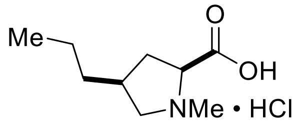

(*4S*)-1-methyl-4-propyl-L-proline
(hydrochloride salt)

(*2S*,*4S*)-1-methyl-4-propylpyrrolidine-2-carboxylic acid hydrochloride:

^{1}H NMR (500 MHz, D_2O) δ 4.19 (dddd, J = 9.9, 8.1, 3.7, 1.7 Hz, 1H), 3.51 – 3.33 (m, 2H), 2.97 (s, 3H), 2.72 (dt, J = 13.6, 7.5 Hz, 1H), 2.62 (p, J = 7.7 Hz, 1H), 1.90 – 1.75 (m, 1H), 1.47 – 1.37 (m, 2H), 1.36 – 1.27 (m, 2H), 0.89 (t, J = 7.3, 3H).

$^{13}C\{^{1}H\}$ NMR (126 MHz, D_2O) δ 171.6, 69.5, 60.8, 41.5, 36.0, 35.0, 34.5, 20.3, 13.1.

IR ν 2925, 1745, 1362, 1191, 772 cm^{-1}.

HRMS (ESI) m/z = $[M + Na]^+$ Calcd $C_9H_{17}NO_2Na^+$ 194.1157. Found 194.1154 (1.5 ppm error).

$[\alpha]_D^{21}$ = -51.60 (*c* 1.0 g/100 mL, H_2O).

(4*S*)-1-methyl-4-propyl-L-proline • HCl (D_2O, ^1H NMR: 500 MHz, $^{13}C\{^1H\}$ NMR: 126 MHz)

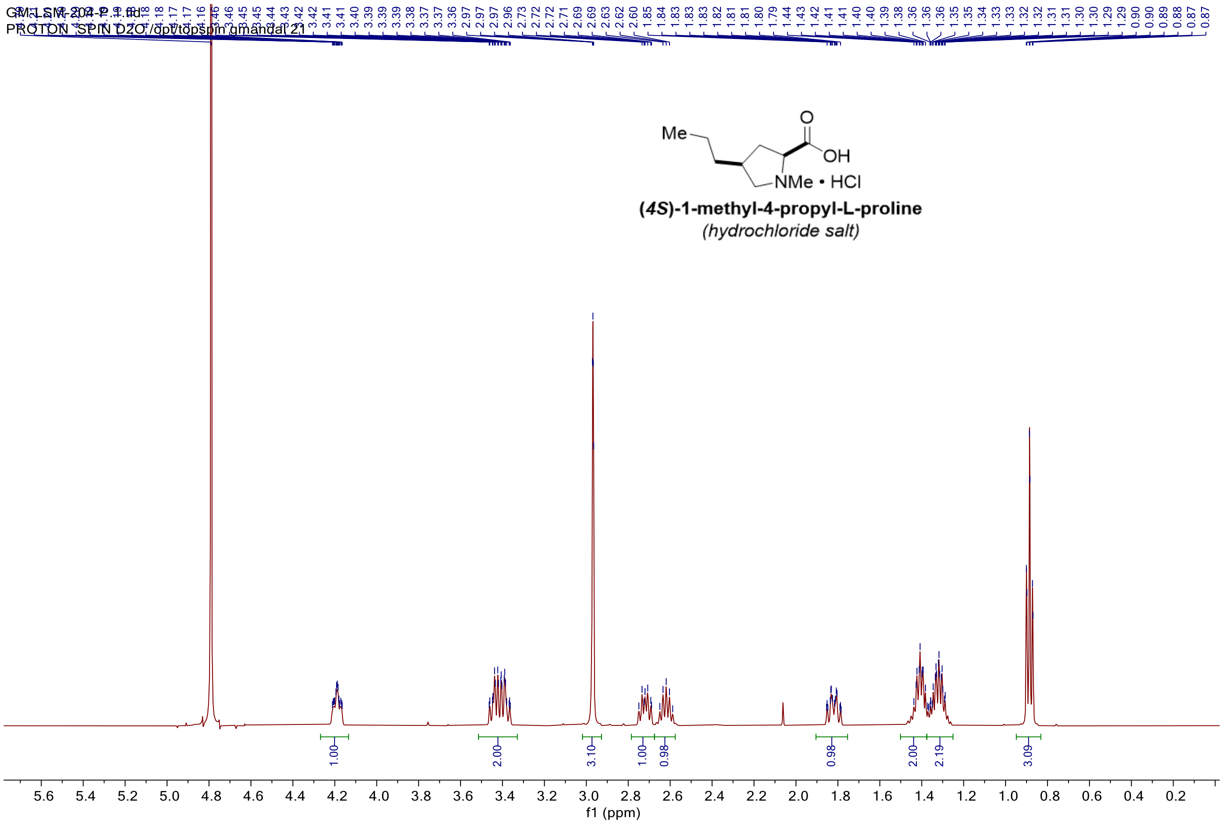

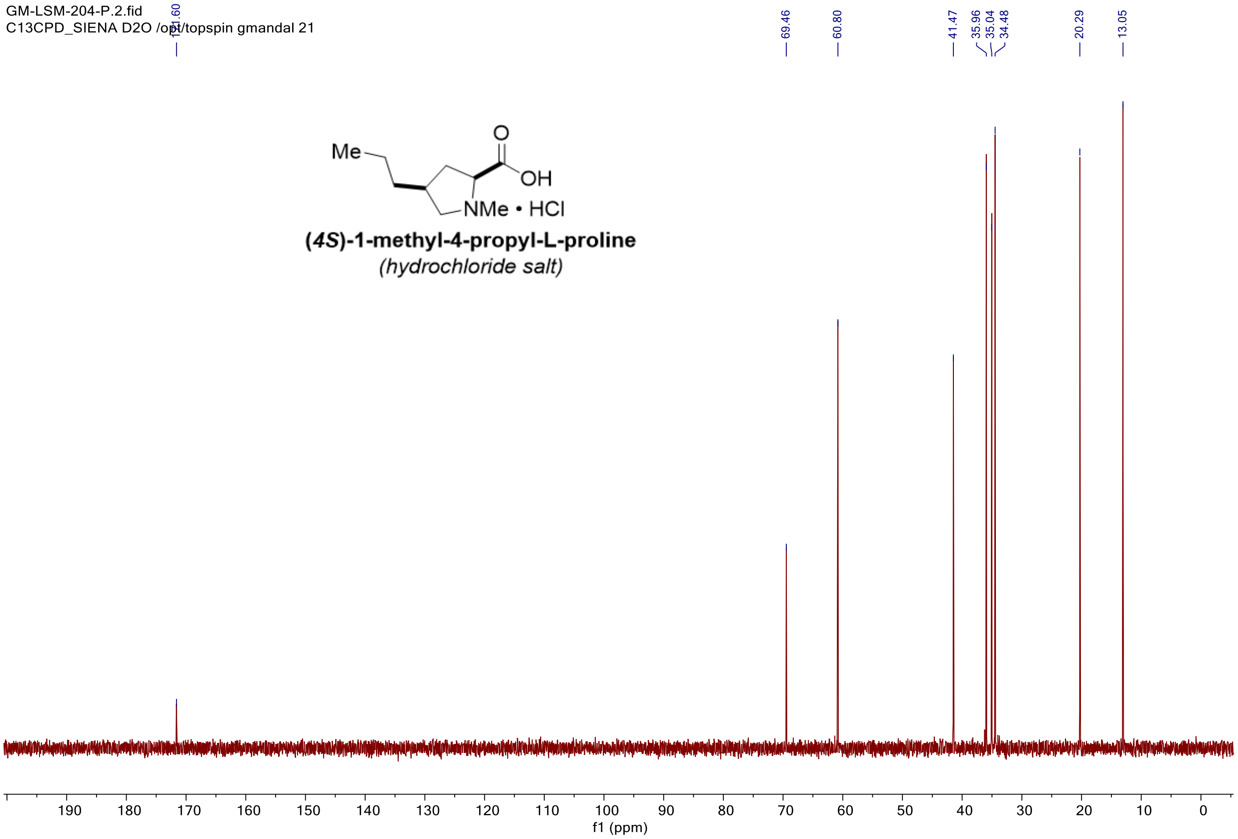

IR

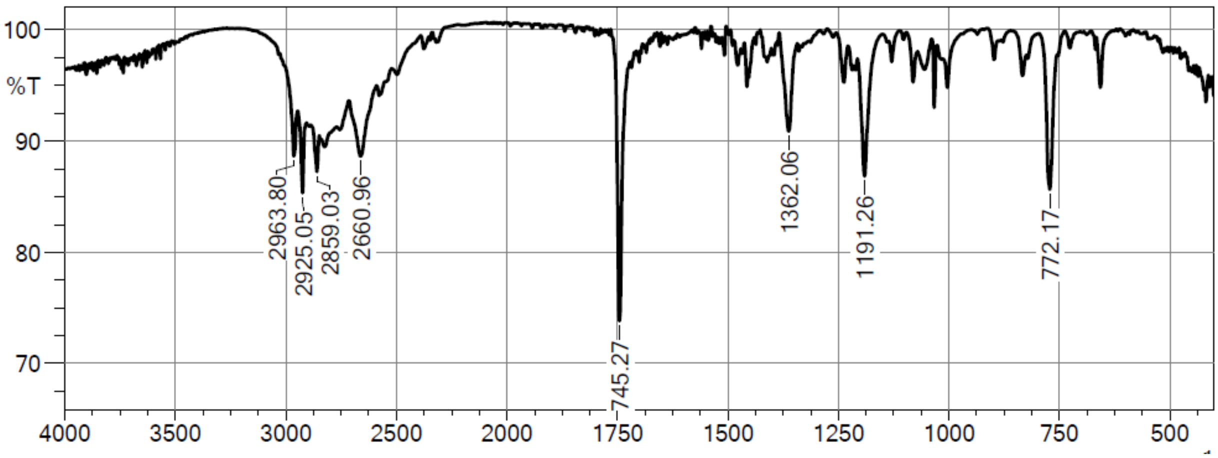
100
%T
90
80
70
2963.80
2925.05
2859.03
2660.96
1745.27
1362.06
1191.26
772.17
4000
3500
3000
2500
2000
1750
1500
1250
1000
750
500

HRMS

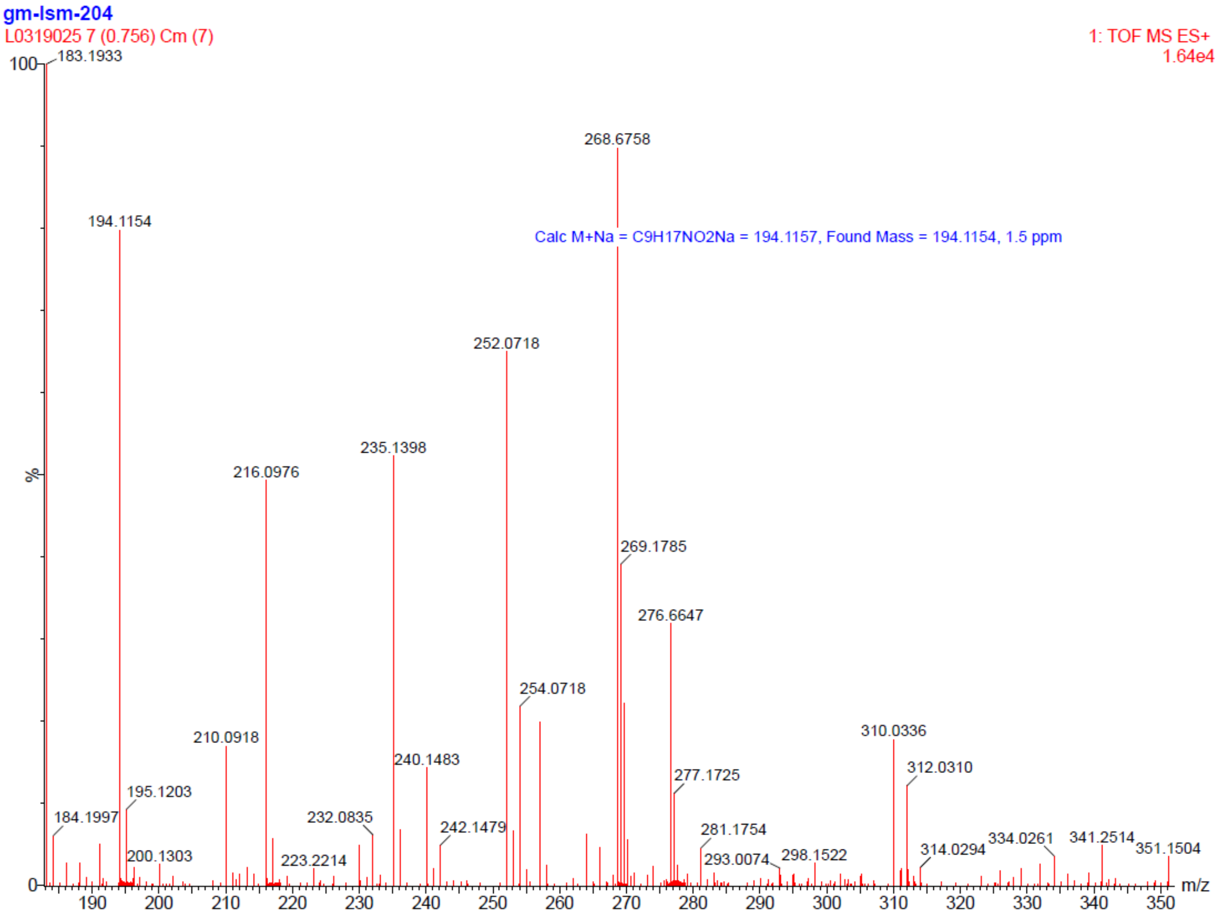
gm-lsm-204
L0319025 7 (0.756) Cm (7)
1: TOF MS ES+
1.64e4
Calc M+Na = C9H17NO2Na = 194.1157, Found Mass = 194.1154, 1.5 ppm
183.1933
268.6758
194.1154
252.0718
235.1398
216.0976
269.1785
276.6647
254.0718
210.0918
310.0336
240.1483
312.0310
277.1725
195.1203
184.1997
232.0835
242.1479
281.1754
200.1303
223.2214
293.0074
298.1522
314.0294
334.0261
341.2514
351.1504
%
m/z

III. X-ray Crystallographic Data

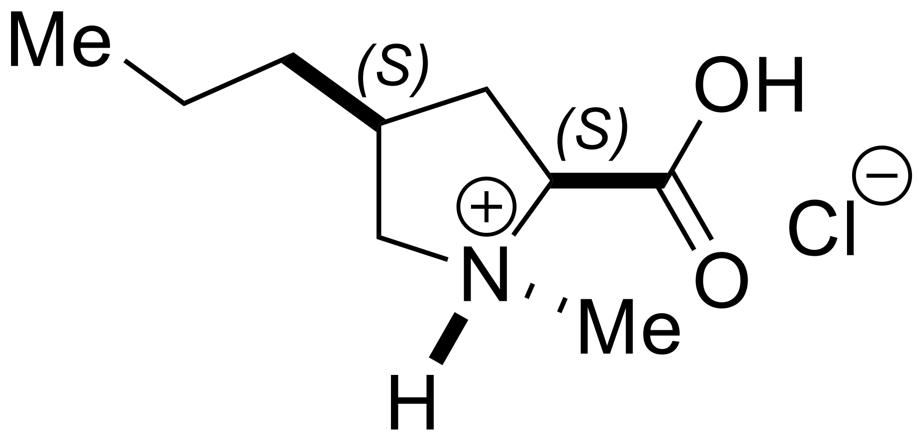

(1*R*,2*S*,4*S*)-2-carboxy-1-methyl-4-propylpyrrolidin-1-ium chloride

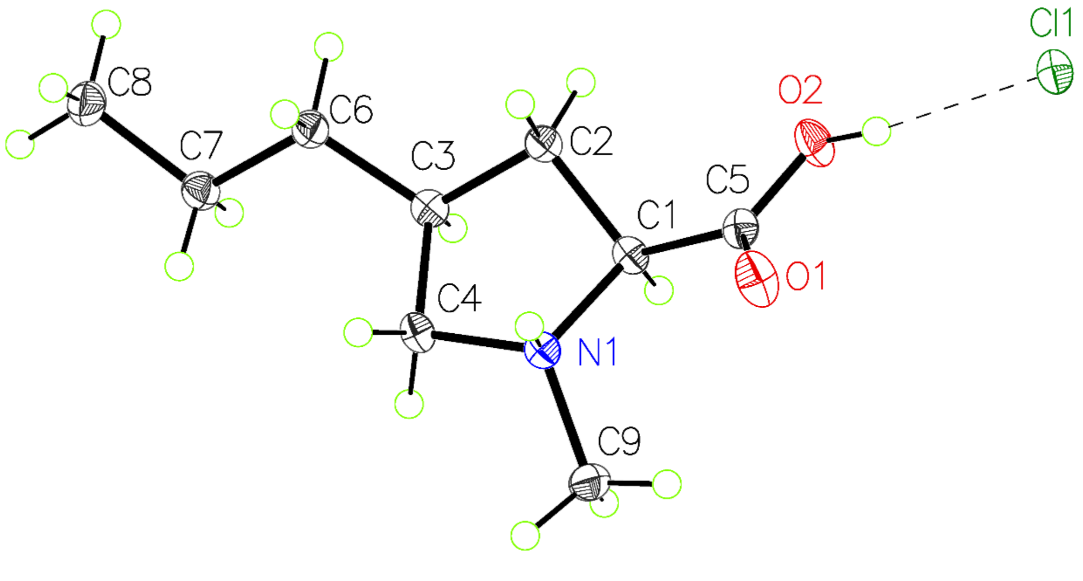

Labeled 50% probability ellipsoid plot of formula unit of GM-LSM-204-P. Crystals were grown by slow evaporation from ethanol. The CCDC number is **2440605**.

Single Crystal X-ray Diffraction Methodology: Single crystal X-ray diffraction data was collected on a Rigaku Synergy 4-circle diffractometer equipped with a Hypix photon-counting detector using Cu-Kα radiation from a microfocus source (Rigaku Americas, The Woodlands, TX). The crystal was cooled to the collection temperature under a stream of cold N_2 using a Cryostream 1000 cryostat (Oxford Cryosystems, Oxford, UK). Data was collected using shutterless scans with 0.5° frame widths and variable scanning rates. Data collection, unit cell determination, data reduction absorption correction, and scaling were done using Rigaku CrysAlisPro.[1] The structure was solved by direct methods using SHELXS[2] and refined by full matrix least squares refinement against F^2 using SHELXL v.2019/3.[3] Olex2 was used as a graphical interface for model building and structure visualization.[4] Non-hydrogen atoms were located from the difference map and refined anisotropically. Hydrogen atoms coordinates were allowed to refine while thermal parameters were constrained to ride on the carrier atoms. Short contact analyses were performed using CCDC Mercury.[5]

Table S1. Crystal data and structure refinement for (*4S*)-1-Methyl-4-Propyl-L-Proline.

Empirical formula	$C_9H_{18}NO_2Cl$	
Formula weight	207.69	
Temperature	100(2) K	
Wavelength	1.54178 Å	
Crystal system	Orthorhombic	
Space group	$P2_12_12_1$	
Unit cell dimensions	a = 6.95128(10) Å	α= 90°.
	b = 11.9403(2) Å	β= 90°.
	c = 12.8885(2) Å	γ = 90°.
Volume	1069.76(3) Å^3	
Z	4	
Density (calculated)	1.290 Mg/m^3	
Absorption coefficient	2.932 mm^{-1}	
F(000)	448	

Crystal size	0.568 x 0.048 x 0.042 mm^3
Theta range for data collection	5.049 to 80.072°.
Index ranges	-6<=h<=8, -15<=k<=15, -16<=l<=16
Reflections collected	30369
Independent reflections	2302 [R(int) = 0.0675]
Completeness to theta = 67.679°	100.0 %
Absorption correction	Analytical
Max. and min. transmission	1.00000 and 0.63733
Refinement method	Full-matrix least-squares on F^2
Data / restraints / parameters	2302 / 0 / 172
Goodness-of-fit on F^2	1.058
Final R indices [I>2sigma(I)]	R1 = 0.0240, wR2 = 0.0627
R indices (all data)	R1 = 0.0247, wR2 = 0.0643
Absolute structure parameter	-0.001(6)
Extinction coefficient	n/a
Largest diff. peak and hole	0.295 and -0.196 e.$Å^{-3}$

References:

1. CrysAlisPro, version 1.171.43.124a, Rigaku Oxford Diffraction, Tokyo, Japan, 2024.
2. Sheldrick, G. M. SHELXS, v.2013-1, 2013.
3. Sheldrick, G. M. *SHELXT* – Integrated space-group and crystal-structure determination. *Acta Cryst. Sect. A: Found. Adv.* **2015**, *71*, 3-8.
4. Dolomanov, O.V.; Bourhis, L.J.; Gildea, R.J.; Howard, J.A.K.; Puschmann, H. *OLEX2:* A complete structure solution, refinement, and analysis program. *J. Appl. Cryst.* **2009**, *42*, 339-341.
5. Macrae, C. F.; Bruno, I. J.; Chisholm, J. A.; Edgington, P. R.; McCabe, P.; Pidcock, E.; Rodriguez-Monge, L.; Taylor, R.; van de Streek, J.; Wood, P. A. Mercury CSD 2.0 – New features for the visualization and investigation of crystal structures. *J. Appl. Cryst.* **2008**, *41*, 466-470.
